# Revealing the Folding of Single-Chain Polymeric Nanoparticles at the Atomistic Scale by Combining Computational Modeling and X-ray Scattering

**DOI:** 10.1021/acsmacrolett.5c00065

**Published:** 2025-03-18

**Authors:** Stefan Wijker, David Dellemme, Linlin Deng, Bence Fehér, Ilja K. Voets, Mathieu Surin, Anja R. A. Palmans

**Affiliations:** † Laboratory of Macromolecular and Organic Chemistry, Institute for Complex Molecular Systems (ICMS), Department of Chemical Engineering and Chemistry, Eindhoven University of Technology, Eindhoven 5600 MB, The Netherlands; ‡ Laboratory for Chemistry of Novel Materials, Center of Innovation and Research in Materials and Polymers (CIRMAP), University of Mons - UMONS, Place du Parc 20, B-7000 Mons, Belgium; § HUN-REN-SU Nanobiophysics Research Group, HUN-REN-SU Biophysical Virology Research Group, and Institute of Biophysics and Radiation Biology, Semmelweis University, 1094 Budapest, Hungary; ∥ Laboratory of Self-Organizing Soft Matter, Department of Chemical Engineering and Chemistry, and Institute for Complex Molecular Systems, Eindhoven University of Technology, P.O. Box 513, 5600 MB Eindhoven, The Netherlands

## Abstract

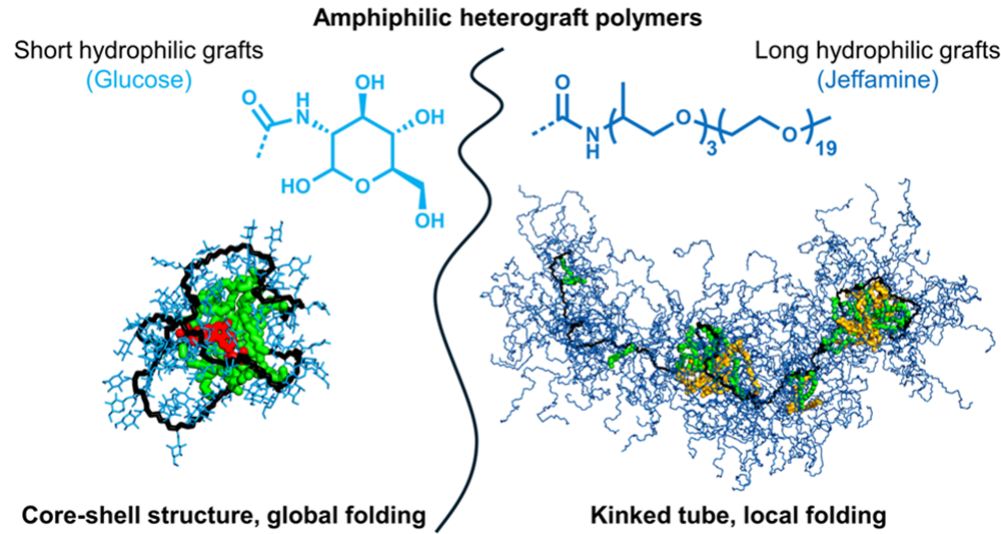

Predicting 3D structures of synthetic heterograft polymers in solution starting from a chemical structure remains a great challenge. Here, we get grip on the 3D structures formed by amphiphilic, random heterograft polymers in water depending on the nature of the hydrophilic graft. Atomistic MD simulations in explicit water on a μs time scale show that large Jeffamine-based grafts combined with randomly distributed hydrophobic grafts induce the formation of worm-like structures with local hydrophobic domains. Replacing Jeffamine by glucose affords core–shell ellipsoidal structures. The simulated small-angle X-ray scattering (SAXS) curves from the simulation results show excellent agreement with experimental SAXS results for the Jeffamine-based copolymers. For the glucose-based copolymers, the experimental SAXS results also indicated the presence of core–shell structures, albeit that (some) multichain aggregation was present. Our work highlights that global conformations of very large heterograft polymers (up to ∼30,000 atoms) can now be studied with (accelerated) MD simulations at the atomic scale in solvent (up to 2.5 million atoms). This joint approach constitutes a reliable tool to understand the folding and possible aggregation behavior of heterograft polymers in solution, paving the way toward predictive modeling of nanoparticle structures from a polymer’s chemical structure.

The emerging field of single chain polymeric nanoparticles (SCPNs) aims at obtaining nanometer-sized particles with well-defined global conformations in solution that are reminiscent of the intricate 3D structures formed by proteins.^1−7^ In view of a specific function, control over the conformation of a synthetic polymer chain in solution is typically achieved by introducing intramolecular chemical cross-links,^8−11^ taking advantage of solvophobic effects,^12−14^ and using secondary interactions such as hydrogen bonding,^15−19^ metal–ligand interactions,^20−22^ or host–guest complexation,^23,24^ and combinations hereof.^25−27^ Water is a particularly interesting solvent in this respect, as hydrophobic interactions can induce a collapse of the polymer chain, after which covalent and noncovalent cross-links lock the chain into compact global conformations.^26,28^ Hereby, particles are formed with a hydrophobic inside and a hydrophilic shell.^1,5^ We and others have applied the resulting compartmentalized nanoparticles as catalyst carriers for catalysis in water^29−33^ and complex media.^34,35^ In addition, collapsed nanoparticles have been applied to tune interactions with natural systems such as proteins and membranes,^36−39^ for cellular targeting,^40,41^ and for developing novel contrast imaging reagents.^42,43^

SCPNs show great potential in a variety of (biological) applications but elucidating intricate details of their global and local conformations that define the 3D structure remains challenging. Our initial naive view was that ‘any’ copolymer structure composed of the correct ratio of hydrophilic and hydrophobic grafts forms core–shell structures comprising a single hydrophobic core. However, detailed investigations using scattering techniques brought to light more and more evidence that this is not always the case.^15,16,44,45^ For example, when using large water-soluble side chains such as JeffamineM1000, one can envisage that high graft densities may result in particles that cannot adopt spherical global conformations.^46,47^ Since the relation between the 2D representation of a polymer chemical structure and the 3D structure of the SCPN formed in water is still poorly understood, we here set out to perform atomistic molecular dynamics (MD) simulations. All-atom MD is a computational modeling approach that allows us to study the structure, dynamics, and interactions of (macro)molecular systems in their environment, and hence provides a reliable representation of the size, shape, and global 3D structure of SCPNs in solution. We combine these simulations with detailed small-angle X-ray scattering (SAXS) experiments. This combination provides new insights into the collapse/folding of random heterograft polymer chains into SCPNs and will be highly useful in designing future primary structures of polymers into desired 3D structures.

The work described here is based on (random co)polymers previously prepared by us (Scheme 1, SI, Section 1.1).^26,48^ The polymers have either JeffamineM1000 (J, v, Scheme 1) or glucosamine (G, w) as hydrophilic grafts to impart water solubility, giving rise to p(J) and p(G). JeffamineM1000 is a polyether with a molecular weight of around 1000 g/mol and an average degree of polymerization of 22 (∼19 EO and ∼3 PO units), and has been routinely used by our group. Glucosamine introduces a smaller graft of high hydrophilicity owing to its many hydroxyl groups. In addition to hydrophilic groups, p(J-BD), p(G-D), and p(G-B) also incorporate hydrophobic grafts, giving the polymers an amphiphilic character. P(J-BD) incorporates both dodecylamine (D, x, Scheme 1) grafts and a chiral benzenetricarboxamide derivative (BTA) (B, y, Scheme 1). Dodecyl and BTA grafts induce the formation of hydrophobic domains. BTAs are additionally capable of forming cylindrical helical stacks with preferred handedness via 3-fold hydrogen bonding, imbuing the nanoparticles with structured hydrophobic domains. Among the glucose-based polymers, p(G-D) and p(G-B) incorporate dodecyl and BTA as hydrophobic grafts, respectively. In addition, all p(G)-based polymers incorporate 1% of Nile Red grafts (z, Scheme 1), which were used in our previous work to track the polymers as well as to report on the local hydrophobicity of the polymer chains via the solvatochromic fluorescence of the hydrophobic Nile Red.^48^ The degree of polymerization (DP) of the polymers lies between 100 and 200. All polymers show global log(P) values of −0.96 or lower, making them hydrophilic in nature and soluble in water (SI, Section 1.2.3).

**Scheme 1:**
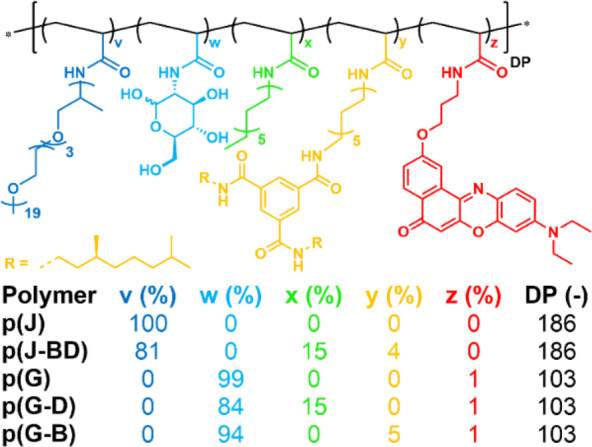
Chemical Structures of the Random Heterograft Polymers

Given the sequenced character and the possible interactions between units, all the atoms need to be taken into account explicitly. Therefore, we decided to perform MD simulations at the atomistic scale. While a coarse grain modeling approach has been successfully applied before and is computationally faster,^28,49,50^ it misses the information at the atomic level, such as hydrogen bonds between different units or with the water solvent, which is important for the systems investigated here. Thus, MD simulations were performed for each polymer as an isolated single chain in explicit water boxes, starting from fully extended conformations and simulated on the 2 μs time scale. Three independent simulation replicas were performed for each system with the AMBER package, using parameters coming from the General Amber Force Field (GAFF) 2.1 to describe the polymers (see details in SI).^51,52^ Although initially built to simulate organic molecules and be compatible with biomolecular force fields in the AMBER package, GAFF has been updated and used by us and others to simulate the folding of heterograft, sequence-defined, and supramolecular polymers.^38,53−56^ GAFF 2.1 has been parametrized for chains containing simple monomer units such as ethylene oxide (as in Jeffamine), amides, propylene oxide, hydrocarbons, as well as substituted benzene units as in BTA.^52,57−59^ The torsions inside the glucose cycles were also verified, to ensure that their conformations were relevant.

P(J-BD) was simulated as random (*r*), block (*b*), and multiblock (m*b*) polymer chains to mimic the dispersity in graft distribution intrinsic to the synthesis of the polymer. These structures are denominated as p(J-*r*-BD), p(J-*b*-BD) and p(J-m*b*-BD), respectively. The p(G-D) and p(G-B) systems were only simulated as random copolymers. The final MD structures of each system are shown in Figure 1 and their sequences are represented as colored bars (see Figure S8 for all MD simulations). All systems were properly equilibrated after 2 μs of simulation, as indicated by the convergence of their root-mean-square deviations (RMSD) values (Figure S9). The results from the simulations show that different morphologies are obtained for the different polymers. This depends on two main parameters, namely the nature of the hydrophilic grafts and the presence/absence of hydrophobic groups. The hydrophilic polymers, p(J) and p(G), both adopt worm-like structures (see left frame of Figure 1). Both chains coil but do not fold into a compact globular structure. They reach a radius of gyration (*R*_G_) of around 10 and 3 nm for p(J) and p(G), respectively

**Figure 1:**
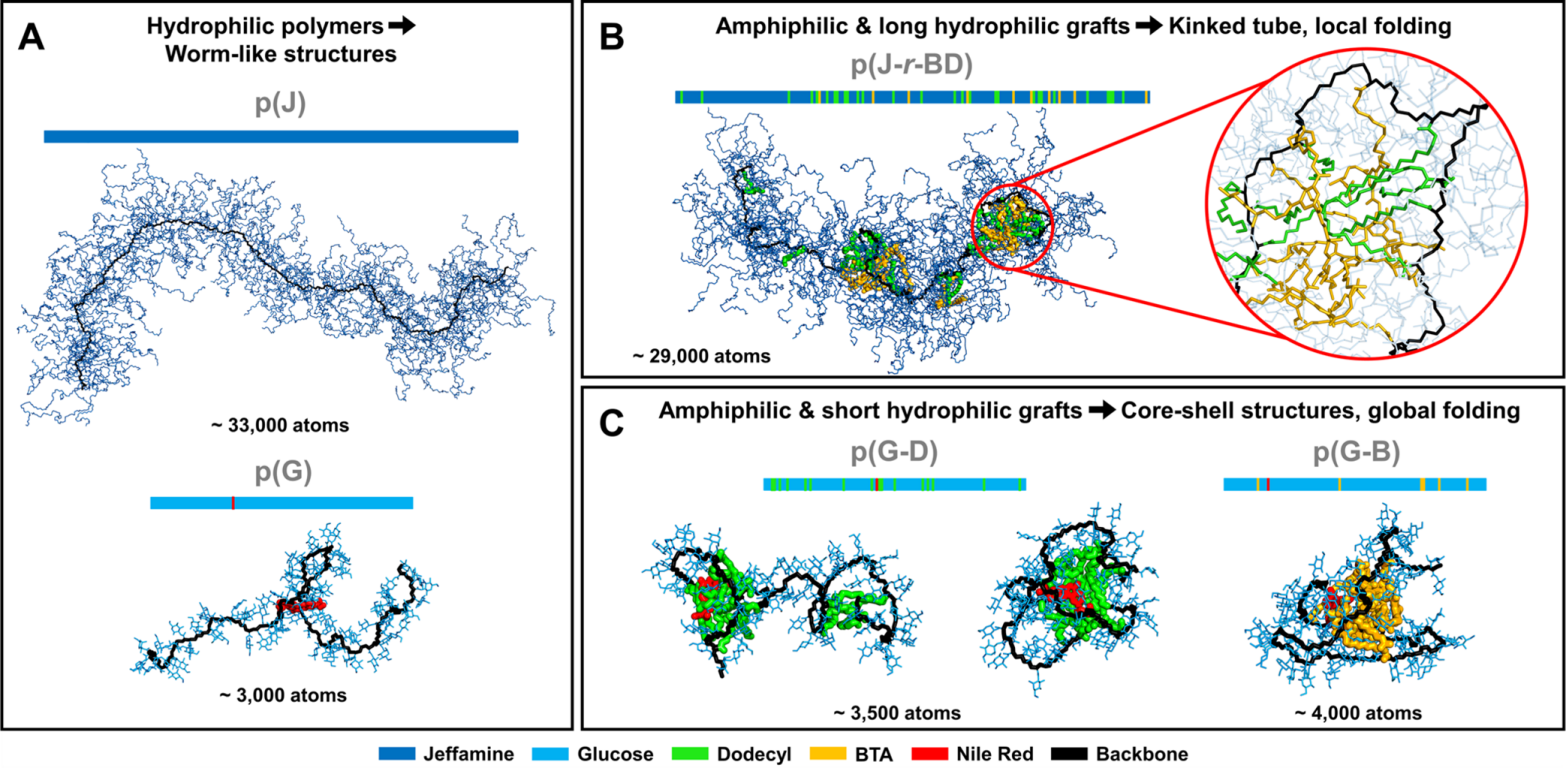
Snapshots of the final conformations obtained during the MD simulations. The number of atoms in each system is written below the MD snapshots and the sequence of monomers is represented as a colored bar (see bottom legend). The backbone is displayed in black. (A) Fully hydrophilic p(J) and p(G) polymers. (B) Jeffamine-based amphiphilic copolymer with a random sequence of monomers, p(J-*r*-BD). (C) Glucose-based amphiphilic copolymers, p(G-D) and p(G-B). Two snapshots are shown for p(G-D): they were obtained after 2 μs of classical MD (left) and after 300 ns of accelerated MD (right).

In the Jeffamine-based copolymers, the introduction of hydrophobic grafts leads to the formation of smaller, more compact structures (Figure S10) for the three different microstructures. P(J-*r*-BD) remains relatively extended (*R*_G_ ≈ 6 to 8.5 nm), but shows local folding around the hydrophobic groups (see inset top frame of Figure 1). The information input in the primary structure is retained in the conformation: units that are far in the sequence remain far in the 3D structures. Also, hydrophobic units close to each other in the sequence are able to merge into the same cluster, but do not meet units at the other end of the copolymer. Over the course of the simulation, multiple, local hydrophobic pockets form along the chain (see top frame of Figure 1). In contrast, p(J-*b*-BD) copolymers contain a single central hydrophobic pocket which does not split into smaller clusters, and the p(J-m*b*-BD) systems exhibit three clusters, which never merge during the simulations.

Inversely, the glucose-based copolymers, p(G-D) and p(G-B), collapse into core–shell nanoparticles (*R*_G_ ≈ 2 nm) (Figure S11). The glucose residues form a shell around a hydrophobic core comprising the dodecyl or BTA grafts, and the Nile Red moiety. This can also be inferred from the significant decrease in solvent-accessible surface area (SASA) of Nile Red during the simulations, which indicates a reduction of Nile Red exposure to water during chain folding (Figure S12). The presence of Nile Red in hydrophobic compartments in the p(G-D) and p(G-B) systems was also detected experimentally (Figure S1), in agreement with the simulations.^60^ The folding of the backbone in p(G-D) and p(G-B) allows hydrophobic units that are far in the sequence to become spatially close in the 3D structure, and the polymers collapse into compact globules. Inside these globules, the backbone dynamics are strongly reduced, as indicated by the sharp decrease of the root-mean-square fluctuations (RMSF) values for the backbone atoms of p(G-D) and p(G-B) upon folding (Figure S13). The dihedral angles’ fluctuations along the backbone are also significantly reduced, showing that the conformational space is reduced as the polymer collapses into a core–shell structure (Figure S14). Such trends have been observed in other folded amphiphilic copolymers as well.^38^ The compact p(G-D) and p(G-B) conformations are further stabilized by intramolecular hydrogen bonds that increase in number over time, while these remain constant for p(G) (Figure S15). The collapse of p(G-D) and p(G-B) is reminiscent of the early stages of protein folding, characterized by nonspecific and local interactions between side-chains,^61,62^ increasing backbone rigidity,^63^ and peptide hydrogen-bond formation.^64^

The copolymers may, however, be trapped for several hundreds of nanoseconds in partly folded states when the hydrophobic units are grouped in two or more clusters (see bottom frame of Figure 1, structure on left). To avoid spending too much time in trapped states, accelerated MD (aMD) simulations are useful.^65^ By applying a boost to the dihedral and potential energy of the system, it becomes easier to escape local minima, thus improving sampling efficiency (full details on the aMD protocol in SI, Section 1.2.6). This methodology was successfully applied on the p(G-D) systems (see structure on the middle, bottom frame of Figure 1), reaching the folded structure after 300 ns of aMD, compared to around 2 μs with conventional MD simulations.

For the copolymers comprising Jeffamine-based grafts, the MD results indicate that the hydrophilic grafts prevent global folding of the polymers. The polar Jeffamine units adopt extended conformations in water, as expected for poly(ethylene glycol) chains.^66,67^ One Jeffamine graft makes around 18 H-bonds per conformation with the surrounding water solvent, which is much more than all the other grafts (Figure S16).

The polarity of the Jeffamine chains turned out to be a crucial parameter to take into account in these MD simulations. Initially, the partial charges were computed using the AM1-BCC model,^68^ leading to underestimated charges on the oxygen atoms, and to the formation of unrealistic, compact globules for the Jeffamine-based systems (see Figure S17). It was recently shown that these atomic charges strongly influenced the interactions of polyethers with water.^69^ Therefore, a new set of charges was derived following the more accurate RESP methodology,^70^ resulting in stronger charges on the oxygen atoms, and in more elongated structures. The sensitivity of the system to partial charges exemplifies to which extent small inaccuracies on charge description can lead to wrong predictions on the shape and size of macromolecular structures.

Our simulations indicate that polar Jeffamine grafts prevent global folding into compact structures as the grafts remain extended and interact with many water molecules. Visual inspection of the final conformations obtained during the MD simulations reveal that this folding does not lead to a compact globule but rather a “kinked tube”, as reflected by measurements of the asphericity parameter (Figure S18). Determination of the SASA shows that the exposure of the dodecyl and BTA side chains to water is similar for both the Jeffamine- and glucose-based copolymers (Figure S19). This means that the p(J-BD) chains do not need to globally collapse to efficiently shield their hydrophobic groups.

As a next step, the 3D structures obtained from the MD simulations were used to simulate SAXS curves, which were compared to experimental SAXS measurements. Figure 2A,B shows the experimental and simulated SAXS curves for p(J) and p(J-BD), as well as the form factor fits to the experimental data. Simulated SAXS curves of p(J) and p(J-BD) were computed at different times, and from additional aMD simulations, to ensure a sufficient sampling (Figure S20 and Table S11). Although the two techniques scan the matter at a different scale (with ideal systems with no molar mass dispersity for MD and disperse, heterogeneous mixture of chains with different microstructures for SAXS), the agreement between experimental data and simulated data is remarkable for the Jeffamine-based (co)polymers. The global shape of the experimental scattering curves is in good agreement with that expected for graft polymers with extended conformations forming worm-like chains. The in-depth analysis of the scattering curves (see SI, Section 2.4) reveals that experimentally, p(J) and p(J-BD) do not show significant aggregation and primarily exist as single polymer chains in solution. The experimental data fit well to a form factor model of a worm-like chain. The results show that the particles adopt conformationally stretched chains with limited flexibility. There is one significant difference between the fits of p(J) and p(J-BD), namely that the fitted value for Kuhn length *l*_k_ – a measure for polymer chain flexibility– is much smaller for p(J-BD) (*l*_k_ = 7 nm) than for p(J) (*l*_k_ = 21 nm), suggesting that replacing Jeffamine chains by smaller hydrophobic grafts increases the polymer’s flexibility. Crucially, the scattering curve of p(J-BD) lacks a clear oscillation around *q* = 1 nm^–1^, which indicates that p(J-BD) does not form a defined, single hydrophobic interior as expected in core–shell structures.^14^ All results taken together corroborate that MD simulations reflect the nature of the formed structures well, namely as extended worm-like structures for p(J) and p(J-BD), and the formation of local hydrophobic pockets in p(J-BD).

**Figure 2:**
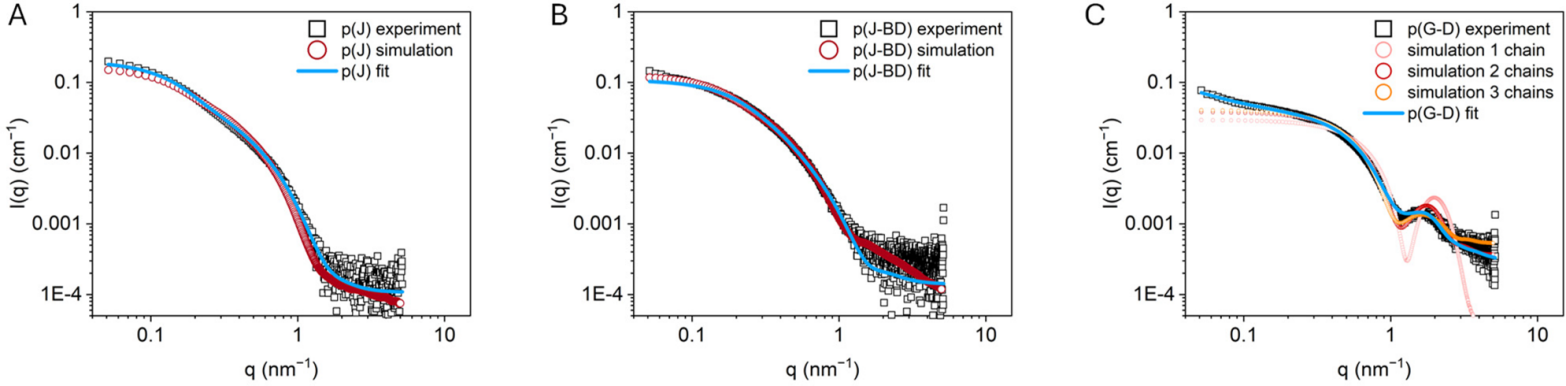
Experimental (black squares) and simulated (open circles) SAXS curves in water. (A) p(J) and (B) p(J-BD) with the worm-like chain form factor fits (blue line) to the experimental data. (C) p(G-D) with a core–shell ellipsoid form factor fit to the experimental data. *c*_pol_ = 1 mg mL^–1^.

Figure 2C shows the experimental and simulated SAXS curves for p(G-D), together with a core–shell ellipsoid form factor fit (data for p(G-B) are shown in Figure S5). The in-depth analysis of the SAXS data is given in SI, Section 2.4. The global shape of the experimental scattering curve indicates that both polymers likely form core–shell nanoparticles of small size, owing to the observed oscillation in the scattering curve around *q* = 1 nm^–1^. The experimental data of p(G-D) fit well to the form factor model of nanometer-sized core–shell ellipsoids. P(G-B) shows a 10 times larger intensity at low *q* compared to p(G-D), suggesting multichain aggregation into larger particles. Interestingly, the simulated SAXS curves for single-chains of p(G-D) and p(G-B) did not match the experimental curves well, although MD results show the formation of core–shell structures. We attributed this discrepancy to the formation of aggregates in solution. To support this hypothesis, mixtures of two chains and three chains were simulated for the p(G-D) copolymer, starting from extended chains (see details in SI and convergence of simulations in Figures S9 and S11). As observed in Figure 2C, the overlap between the experimental and simulated curves is significantly improved when considering multichain aggregates (see also Table S11). A mixture of species, comprising SCPNs but also small aggregates, probably coexist in solution. This would be in line with DLS measurements, which show a wide distribution of sizes for the p(G-D) particles, from about 2 to 10 nm (Figure S2). Aggregation likely occurs in the early steps of folding, before the complete shielding of the hydrophobic moieties. This example demonstrates the robustness of this approach combining MD simulations and SAXS, as it allows to distinguish particles of similar shape, core–shell structures, but of slightly different sizes as well. Such resolution is difficult to attain using experimental means alone.

In conclusion, we have demonstrated that atomistic scale MD simulations constitute a promising tool to gain insight into the folding behavior of amphiphilic heterograft polymers. Indeed, the current computational power using GPUs is now adapted to treat these very large systems (e.g., > 30,000 atoms for p(J) in a box of ∼ 2,500,000 atoms of solvent) at the atomistic scale. The nature of the 3D structures resulting from MD simulations, kinked tubes or core–shell structures, are supported by the experimental SAXS data. Using a combined MD and SAXS approach reveals that Jeffamine-based copolymers form globally extended structures capable of forming local hydrophobic domains. Copolymers functionalized with hydrophilic glucose grafts are instead capable of global collapse into core–shell structures comprising a single central hydrophobic core, but are also prone to form multichain aggregates. We believe that atomistic MD simulations, especially accelerated methodologies, are now at a timely stage to help design sequence-controlled or random heterograft polymers that fold into nanosized structures with desired architectures, before embarking on lengthy synthesis procedures.

## Supporting Information Available

Polymer characterization, MD and SAXS methodology (PDF)
