# Supplementary material for: Revealing the Folding of Single-Chain Polymeric Nanoparticles at the Atomistic Scale by Combining Computational Modeling and X-ray Scattering
Source: ACS Macro Lett. 2025 Mar 18;14(4):428–33. doi: 10.1021/acsmacrolett.5c00065 (PMC12004929; doi:10.1021/acsmacrolett.5c00065)
Supplement: Supplementary file 1 — mz5c00065_si_001.pdf [file mz5c00065_si_001.pdf]

Electronic Supporting Information

Revealing the folding of single-chain polymeric nanoparticles at the atomistic scale by combining computational modeling and X-ray scattering

Stefan Wijker[‡a], David Dellemme[‡b], Linlin Deng[a], Bence Fehér[cd], Ilja K. Voets[d], Mathieu Surin*[b], Anja R.A. Palmans*[a]

[a] Laboratory of Macromolecular and Organic Chemistry, Institute for Complex Molecular Systems (ICMS), Department of Chemical Engineering and Chemistry, Eindhoven University of Technology, Eindhoven 5600 MB, The Netherlands. [b] Laboratory for Chemistry of Novel Materials, Center of Innovation and Research in Materials and Polymers (CIRMAP), University of Mons - UMONS, Place du Parc 20, B-7000 Mons, Belgium.

[c] HUN-REN-SU Nanobiophysics Research Group, Budapest, Hungary; HUN-REN-SU Biophysical Virology Research Group, Budapest, Hungary; Institute of Biophysics and Radiation Biology, Semmelweis University, 1094, Budapest, Hungary.

[d] Laboratory of Self-Organizing Soft Matter, Department of Chemical Engineering and Chemistry, and Institute for Complex Molecular Systems, Eindhoven University of Technology, P.O. Box 513, 5600 MB Eindhoven, the Netherlands.

* To whom correspondence should be addressed
E-mail: Mathieu Surin: mathieu.surin@umons.ac.be; Anja R.A. Palmans: a.palmans@tue.nl
‡ Both authors contributed equally

Table of Contents

Table of Contents 2
1. Materials and methods 3
1.1. Materials 3
1.2. Methods 3
1.2.1. Fluorescence Spectroscopy 3
1.2.2. Densimetry 3
1.2.3. log(P) calculations 3
1.2.4. Dynamic Light Scattering (DLS) 3
1.2.5. Small-angle X-ray Scattering (SAXS) 3
1.2.6. Atomistic scale MD simulations 4
2. Experimental Section 6
2.1. Polymer data summary 6
2.2. Fluorescence Spectroscopy 6
2.3. Dynamic Light Scattering 7
2.4. Small-angle X-ray Scattering 8
2.5. Atomistic scale MD simulations 14
3. References 31

1. Materials and methods

1.1. Materials

Nile Red and phosphate buffered saline tablets were purchased from Merck or Fischer Scientific. Ultrapure water was obtained using a Milli-Q® ultrapure system. The polymers were synthesized previously.[1,2] For the in-depth synthesis procedure and polymer characterization, we refer to the references mentioned. In ref 1, P7 is the same polymer as p(J) in this publication and P3 is p(J-BD). Likewise in ref 2, PDG is p(G-D) and P100BG is p(G-B).

1.2. Methods

1.2.1. Fluorescence Spectroscopy

The Nile Red fluorescence spectra shown in Figure S1 were recorded in previous research as described previously.[1,2] In order to more accurately determine the emission maxima, Lowess smoothing was performed and the emission maxima was chosen as the wavelength corresponding to the maximum emission intensity after smoothing.

1.2.2. Densimetry

The density of polymer solutions was determined using a DMA 4500 M Anton Paar densitometer *via* the oscillating-tube technique. The densitometer was calibrated at 20.00 °C, using air and pure water as reference samples. The measured density was reproducible up to the 5th (last) digit. Each sample was measured twice. A solution of p(J) was measured at a concentration of 1.0 mg mL^{-1} at 20.00 °C. From these measurements, the apparent specific volume of p(J) in water $\bar{v}_{\mathrm{app}}$ (cm^3 g^{-1}) was calculated according to $\bar{v}_{\mathrm{app}} = \frac{1}{d_{\mathrm{s}}} \cdot \left(1 - \frac{d_{\mathrm{p}} - d_{\mathrm{s}}}{c_{\mathrm{p}}}\right)$, with d_{s} the density of the solvent (g cm^{-3}), d_{p} the density of the polymer solution (g cm^{-3}), and c_{p} the polymer concentration (g cm^{-3}). $\bar{v}_{\mathrm{app}}$ was calculated as 0.8415 cm^3 g^{-1}. The apparent specific volume of p(J-BD) was assumed to be equal to that of p(J) due to the large mass fraction of Jeffamine@M1000 in p(J-BD). The apparent specific volumes were not determined for p(G-D) and p(G-B).

1.2.3. log(P) calculations

Values of the partition coefficient of the polymers were calculated using the MarvinSketch 20.11 software by ChemAxon Ltd using the ChemAxon method at an electrolyte concentration of 0.1M NaCl concentration. The log(P) of the polymers were calculated by summation of the log(P) of the monomer building blocks, calculation over the respective acrylamide monomers of the grafts, while ignoring the polymer end-groups. Using this method, the values of log(P_{i}) found were 8.71 for the BTA monomer, 3.00 for the Nile Red monomer, 4.34 for the dodecyl monomer, -2.42 for the glucose monomer, and -2.73 for the Jeffamine@M1000 monomer. The calculation of the polymer log(P) is calculated from the log(P_{i}) the value of the monomer, and the mol fraction of the monomer x_{i} follows:

$$\log P = \sum_i (x_i \log(P_i))$$

1.2.4. Dynamic Light Scattering (DLS)

Dynamic Light Scattering measurements were performed on a Malvern Instruments Zetasizer µV equipped with a λ = 830 nm laser in Sarstedt disposable cuvettes after sample filtration to remove dust at 20 °C. The scattering intensity was recorded at a fixed scattering vector $q = \frac{4\pi n_{\mathrm{solvent}}}{\lambda} \cdot \sin\frac{\theta}{2}$, with n_{solvent} the refractive index of the solvent, θ the scattering angle in degrees and λ the wavelength of the laser in nm. n_{solvent} = 1.335. The scattering intensity was recorded in triplicate, at a scattering angle of 90°, at 13 measurements of 3 seconds each. The fluctuations in the scattering intensity were automatically analyzed by the built-in CUMULANT algorithm to obtain the intensity, volume, and number distributions of dissolved particles. The apparent hydrodynamic radius R_{H} was calculated from the diffusion coefficient of the particles D as $R_{\mathrm{H}} = k_{\mathrm{B}}T/(6\pi\eta D)$, with k_{B} the Boltzmann constant, T the temperature of the solution in K, and η the solvent viscosity. η = 1.0016 mPa s.[3]The final R_{H} was taken from the intensity distribution as the average of the three measurements. The measurements of p(J-BD) were instead recorded on an ALV CGS-3 instrument equipped with an ALV-7004 digital correlator and a λ = 532 nm laser in cylindrical glass cuvettes (i.d. = 0.8 cm) after sample filtration with a Whatman 200 nm PVDF syringe filter to remove dust. The scattering intensity was recorded at scattering angles of 30° to 150° with a step size of 10°, at 6 measurements of 15 seconds per angle. The fluctuations in the scattering intensity were analyzed by applying the CONTIN algorithm to the first order autocorrelation function derived from the trace of the scattering intensity using the After-ALV software by Dullware Inc. to obtain the decay rate Γ of the particles in solution. The diffusion coefficient of the particles D was derived from the slope in a plot of Γ as a function of q^2, from which R_{H} was derived in the same manner as on the Zetasizer.

1.2.5. Small-angle X-ray Scattering (SAXS)

SAXS was measured on beamline BM29 (BioSAXS) at ESRF.[4] The scattering intensity $I(q)$ was recorded as a function of the scattering vector q over the range 0.05 – 5 nm^{-1} using monochromatic X-rays with an energy of 12.5 keV as recorded on a

Pilatus3 2M detector in vacuum. Here, $q = \frac{4\pi}{\lambda} \cdot \sin\frac{\theta}{2}$, with λ the X-ray wavelength (0.1 nm) and θ the scattering angle in degrees. Samples were measured in a quartz glass capillary with a diameter of 1 mm at 20 °C using the sample changer mode. Samples were measured for 10 frames of 1 second each under flow to minimize radiation damage. Standard corrections were applied automatically at ESRF (empty capillary and solvent contributions, absolute scale corrections, *etc.*). The scattering intensity on absolute scale is a function of the weight-averaged molecular weight of the particles M_w (g mol^{-1}), the weight concentration of particles c (g cm^{-3}), the scattering length density difference per mass between particle and solvent $\Delta\rho_M$ (cm g^{-1}), Avogadro's constant N_A (= $6.0224 \cdot 10^{23}$ mol^{-1}), the form factor $P(q)$ describing intraparticle interference, the structure factor $S(q)$ describing interparticle interference, and incoherent background scattering as

$$I_{\mathrm{abs}}(q) = M_{\mathrm{w}} \cdot c \cdot \Delta\rho_{\mathrm{M}}^2 \cdot N_{\mathrm{A}}^{-1} \cdot P(q) \cdot S(q) + background.$$

The scattering length density difference per mass between particle and solvent $\Delta\rho_M$ was calculated as

$$\Delta\rho_{\mathrm{M}}^2 = (\rho_{\mathrm{polymer}} - \rho_{\mathrm{solvent}})^2 \cdot \bar{\nu}_{\mathrm{app}}^2.$$

Here ρ_{polymer} is the scattering length density of the polymer, ρ_{solvent} is the scatter length density of the solvent, and $\bar{\nu}_{\mathrm{app}}$ (cm^3 g^{-1}) is the apparent specific volume of the particles in solution. The scattering length density was calculated from the electron density of the solvent and particle respectively. The scattering length density of water, the solvent, was calculated as $9.47 \cdot 10^{10}$ cm^{-2} and the scattering length density of p(J) and p(J-BD) were calculated as $11.01 \cdot 10^{10}$ cm^{-2}. At low concentrations, the interparticle interference is negligible, and $S(q) \approx 1$. The angular dependence of the scattering curve is therefore described by the form factor only. At sufficiently low scattering vector for small particles, we probe the entire particle, and the scattering curve reaches a plateau, corresponding to $P(q) \approx 1$. We can then calculate the theoretical scattering intensity of our sample at a scattering angle of 0°, $I(0)_{\mathrm{th}}$, using the above equation by substituting $P(q) = 1$ and $S(q) = 1$. $I(0)$ can also be determined from the scattering curve in tandem with the radius of gyration R_G *via* the shape-independent Guinier analysis according to $I(q) \approx I(0)_{\mathrm{exp}} \cdot e^{-q^2 R_G^2/3}$, in which $\ln(I(q))$ is plotted against q^2. A linear fit of the scattering data and extrapolation to $q = 0$ yields $I(0)_{\mathrm{exp}}$ as the y-intercept at $q = 0$, and R_G from the slope. The maximum q included in the Guinier analysis was constrained to $qR_G < 1.3$, as the Guinier analysis does not hold for larger values. Datapoints at low q that show a clear upturn were not used for the fit. From this, the weight-averaged number of polymer chains per particle is calculated according to $N_{\mathrm{agg}} = I(0)_{\mathrm{exp}}/I(0)_{\mathrm{th}}$. Form factor fits were performed in SASfit.[5] p(J) and p(J-BD) were fit using a factorized approach for long, thin objects as $I(q) = P'(q)P_{cs}(q) + bg$, with $P'(q)$ the form factor of the long dimension, $P_{cs}(q)$ the form factor of the cross-section, and bg a constant background term. The Worm PS3 function without excluded volume effects was chosen for $P'(q)$, which describes the scattering objects as worm-like chains (see https://sasfit.org/#the-manual, page 371, section 9.2.4.7). The cross-section term $P_{cs}(q)$ was described as a Boucher cylinder cross-section (see https://sasfit.org/#the-manual, page 343, section 9.2.2.5), to mirror the decrease in radial density of the chains at larger radius values as the grafts stretch out into the solvent. From this, the length of the scattering objects L, the Kuhn length l_k, the cross-sectional radius R_{cs}, and the distribution width of the cross-sectional radius σ_{cs} were determined as global parameters using a simultaneous fitting procedure to 5 different concentrations (c_{pol} = 0.5 – 2.5 mg mL^{-1}) in which chi-squared χ^2 was minimized using the method of least squares. The shape parameter α was a fixed input parameter equal to the slope in a log-log plot of the scattering intensity $I(q)$ against q between q = 0.8 – 1.2 nm^{-1} (see ESI Section 2.4). p(G-D) and p(G-B) were fit using two additive distributions as $I(q) = P_1(q) + P_2(q) + bg$, with $P_1(q)$ the form factor for ellipsoidal core-shell structures (see https://sasfit.org/#the-manual, page 461, section 9.6.4) to describe the small nanoparticles in solution, and $P_2(q)$ a power law to fit the observed scattering at low q originating from a small number of larger aggregates. $P_2(q)$ was chosen as the generalized Gaussian coil form factor with R_G set outside the experimental range to model a single power law (see https://sasfit.org/#the-manual, page 418, section 9.4.1.5). From this, the polar radius R_{polar} and the equatorial radius R_{eq} of the ellipsoid were determined, in addition to the thickness of the hydrophilic shell R_{shell} and the power law scaling x^{-1} (equal to the Flory exponent ν in the generalized Gaussian coil model). Similar to before, these parameters were determined as global parameters using a simultaneous fitting procedure to 5 different concentrations (c_{pol} = 0.5 – 2.5 mg mL^{-1}) in which chi-squared χ^2 was minimized using the method of least squares. In this additive model, the program did not accurately represent the uncertainty on the fit parameters, so the fitting procedure was repeated separately or each concentration, and the averaged parameter values with uncertainty are shown in Table S2 underneath the simultaneous fit parameters labelled as '-averaged'. R_G was fixed to 250 nm, such that the generalized Gaussian coil model regresses to a simple power law within the experimental q-regime. For the contrast terms of the shell, solvent, and core in the core-shell ellipsoid form factor model, $\varepsilon_{\mathrm{shell}} = 1$, $\varepsilon_{\mathrm{solvent}} = 0$, and $\varepsilon_{\mathrm{core}} = -1$, respectively.

1.2.6. Atomistic scale MD simulations

MD simulations were carried out using the AMBER package.[6] The polymer chains were assembled in several steps. First, the monomeric units and chain-ends were built individually with the Avogadro 1.2.0 software.[7] Each of these residues was then assigned atomic partial charges following the RESP methodology, using the antechamber module of AMBER.[8] The quantum calculations were done with the Gaussian 16 software.[9] Note that partial charges previously assigned with the AM1-BCC methodology,[6] led to unsatisfactory results, see Figure S17. The polymer chains were then built by assembling the monomers in the desired sequence, with randomized chirality, using the *sequence* command of the LEaP module of AMBER. The ratio between the α and β anomers of the glucosamine monomers was set as 60 % α and 40 % β, as measured experimentally in aqueous solution.[10] Three different sequences were investigated for the p(J-BD) copolymers, to study the effect of the primary structure on the 3D conformations. The first one is a random copolymer, denominated as p(J-*r*-BD). The second one is a bloc

copolymer, denominated as p(J-*b*-BD), in which all dodecyl and BTA side-chains are placed consecutively in the center of the chain. The third one is a multiblock copolymer, p(J-m*b*-BD). The dodecyl and BTA grafts are distributed in three clusters, at the beginning, the middle and the end of the copolymer. The glucose-based copolymers, p(G-D) and p(G-B), were only studied as random copolymers. Each polymer was simulated in triplicate, identified by the Roman numerals I, II and III. For the Jeffamine-based systems, the same sequence was used for all three replicates, i.e., p(J-*r*-BD) I, II and III all have the same sequence of monomers. For the glucose-based systems, a new (random) sequence was inputted for each replicate. All the force-field parameters for the polymers and their side-chains (Jeffamine, glucose, dodecyl, BTA and Nile Red) are parameters from GAFF 2.1.[11] The starting structure of the polymer chains were reworked by hand to remove most of the steric clashes using the PyMOL software, which was also used to visualize all the MD snapshots.[12] This step was followed by a geometry optimization in implicit solvation, with 1,000 steps of steepest descent followed by 9,000 steps of conjugated gradients. The stable molecules were then solvated in rectangular water boxes, ensuring a minimal distance between any solute atom and the edge of the box of 25 and 40 Å for the Jeffamine- and glucose-based systems, respectively. One Na^+ ion was added to bring the system to electroneutrality. The OPC3 water model was used to describe the solvent.[13] The hydrogen mass repartitioning (HMR) scheme was applied on all solute atoms, enabling the use of a timestep of 4 fs.[14] All subsequent simulations were performed with the GPU version of AMBER.[15] The MD protocol followed five steps. First, a 10,000 steps minimization (1,000 steps of steepest descent and 9,000 steps of conjugated gradient) was carried out on the solvent molecules and ion only, using positional restraints on the solute with a force constant of 25 $kcal.mol^{-1}.Å^{-2}$. A second minimization step was carried out without restraints, with the same methodology. Then, the system was heated in 1 ns from 10 to 300 K in the NVT ensemble, with 1 more ns of equilibration under these conditions. During the heating step, positional restraints were applied on the solute atoms with a force-constant of 10 $kcal.mol^{-1}.Å^{-2}$. The temperature was maintained at 300 K with a Langevin thermostat, using a collision frequency of 1 ps^{-1}. The system was then equilibrated for 10 ns in the NPT ensemble. The pressure was maintained at 1 bar with a Monte-Carlo barostat, and the pressure relaxation time was set at 2 ps. Finally, the production phase of the simulation was launched in the same conditions for 2 µs. This portion of the simulation was analyzed, saving a snapshot each ns. For all these steps, the cutoff for non-bonded interactions was fixed at 8.0 Å and the long-range electrostatic interactions were treated by the particle-mesh Ewald method. The SHAKE algorithm was applied to constrain bonds involving hydrogen atoms. Note that the simulations on the Jeffamine polymers were restarted after 1.2 µs: the last snapshot was extracted and re-solvated in a smaller solvent box, and the simulation was extended until 2 µs, such as to save computational time.

Accelerated MD (aMD) simulations were performed for 400 ns on the p(J) and p(J-r-BD) systems (starting from the snapshot extracted after 1.2 µs) and for 300 ns on the p(G-D) copolymer (starting from the initial extended conformation).[16] The building of the system and the first four steps of the simulations, before the production phase, followed the protocol described above. However, the HMR scheme was not applied and the timestep was set to 2 fs. The p(G-D) aggregates of two or three chains were simulated with the same aMD protocol, for more than 1 µs. The macromolecules started as fully extended chains, with initial intermolecular contacts between some dodecyl moieties, such as to promote intermolecular assembly instead of single-chain folding.

The basic principle of aMD is to provide a boost on the energy when the system reaches stable states, to facilitate transitions between local minima separated by high energy barriers. Here, two boosts were applied: one on the dihedral energy, and one on the potential energy. They depend on two boost parameters, E and α, which were determined as follows for the dihedral energy:

$$E_D = (4\ x\ N_{residues}) + E_{dihed,\ avg}$$
$$\alpha_D = (0.8\ x\ N_{residues})$$

With E_D and α_D, the dihedral boost parameters, $N_{residues}$, the number of solute residues and $E_{dihed,avg}$, the average dihedral energy, measured over the 10 ns of equilibration in the NPT ensemble. Similarly, the boost parameters for the potential energy, E_P and α_P:

$$E_P = (0.2\ x\ N_{atoms}) + E_{pot,\ avg}$$
$$\alpha_P = (0.2\ x\ N_{atoms})$$

With N_{atoms}, the total number of atoms in the system (including solvent) and $E_{pot,\ avg}$, the average potential energy, measured during the 10 ns of equilibration in the NPT ensemble. Note that the boost parameters may be adapted for a higher or lower acceleration.

After the simulations, all analyses were done with the *cpptraj* module of Amber.[17] The root-mean-square deviations (RMSD) values were computed after removal of the translational and rotational movements, taking the first snapshot of the production phase as the reference structure. The radius of gyration (R_G) is a measure of compactness and gives the average distance of an atom to the geometric center of the system. The R_G was measured on all atoms except hydrogens. The solvent-accessible surface area (SASA) measures the exposure of a group of atoms to its surrounding environment. The higher the SASA, the more the moiety is exposed. SASA values were calculated with the LCPO algorithm, using a van der Waals radius of 1.4 Å for the solvent probe (i.e., the radius of a water molecule). Root-mean-square fluctuations (RMSF) are an indicator of the mobility of an atom or group of atoms. The higher the RMSF, the greater the positional fluctuations. First, translational and rotational movements were suppressed by aligning all structures to a reference, generally the first conformation of the production phase (for the RMSF calculated in the last 400 ns, the reference structure was the first of this time interval). Then, RMSF values were computed for each monomer on the backbone carbon atom bearing the side-chain. The fluctuations of the

dihedral angles were measured for all bonds in the backbone as their average deviation to their mean value. The fluctuation of one angle θ_A around its mean value θ_{mean}, calculated for the N conformations sampled, was computed as follows:

$$\frac{\sum_i^N |\,[(\theta_{A,i} - \theta_{mean} + 180)\ modulo\ 360] - 180\,|}{N}$$

To avoid the problem of working with a periodic variable, the mean dihedral angle was computed in the cartesian space.[18] Each individual angle, expressed in degrees in the range (-180° ; 180°), is converted in (x,y) coordinates. The average values over the N conformations of the x and y coordinates define the mean dihedral angle in the cartesian space. This angle is then converted back to polar coordinates, in degrees, as θ_{mean}. In the formula, the addition of 180° and the application of modulo 360 are done to ensure that $\theta_i - \theta_{mean}$ values are expressed in the range (0° ; 360°). Then, 180° are subtracted to measure the difference in the desired (-180° ; 180°) interval, and the absolute value is taken, as we are only interested in the absolute difference. The hydrogen bonds were detected with the *hbond* command of cpptraj, with distance and angle cutoffs of 3.0 Å and 135°, respectively. The asphericity parameter, whose value ranges between 0 for a perfect sphere and 1 for rod-like conformations, was computed based on the gyration tensor values, as described elsewhere.[19] The simulated SAXS curves were generated using the CRYSOL 3.2.1 software.[20] The average displaced solvent volume per atomic group, the contrast of the hydration shell and the relative background used to generate the simulated SAXS curves were optimized against the experimental SAXS curves. The discrepancy between the simulated and experimental curves is quantified by CRYSOL with a χ^2 value, which compares, for each data point (each q value), the simulated intensity and the experimental intensity. The higher the χ^2 value, the less the curves overlap. For the p(J) system, three average curves were obtained, at different times: in the range 520 – 700 ns, in the range 1820 – 2000 ns and in the last 100 ns of the accelerated simulation, to ensure that the conformations probed during the simulations remain in agreement with the experimental SAXS spectra over time. 10 conformations of each replicate (p(J) I, p(J) II and p(J) III), one conformation each 20 ns (or each 10 ns for the aMD simulation) were extracted to compute the average curves. Similarly, the scattering curves of p(J-BD) were obtained by averaging over the three replicates of the three sequences, p(J-*r*-BD), p(J-*b*-BD), and p(J-*mb*-BD). The scattering curves of p(G-D) for one, two or three chains were obtained by averaging the spectra obtained for 10 conformations generated through aMD simulations.

2. Experimental and Modeling Section

2.1. Polymer data summary

Table S1 summarizes the polymer properties described in the rest of the ESI. $M_{n,th}$ is the polymer's theoretical molecular weight determined *via* ^{19}F-NMR (nuclear magnetic resonance), the molar mass dispersities ($Đ$) of the backbone, $Đ_{bb}$, and functionalized polymer chain, $Đ_{pol}$, respectively, determined *via* SEC as described in previous publications.[1,2] The polymers have a narrow distribution in backbone dispersity and relatively narrow distribution after functionalization. The dispersity of the glucose-functionalized polymers could not be determined due to limited solubility in organic solvents available for SEC-analysis. log(P) is the partition coefficient of the polymers (ESI Section 1.2.3). All 4 polymers have a low log(P) value of approximately -1 or even lower, indicating that, in general, the polymers are expected to be hydrophilic in nature and will dissolve well in water. λ_{max} is the emission maximum of physically encapsulated Nile Red (p(J) and p(J-BD)[1] or the Nile Red attached to the polymer backbone.[2] The emission spectra are combined into Figure S1. The spectra are interpreted in more detail in ESI Section 2.2. R_H is the hydrodynamic radius of the nanoparticles determined *via* DLS, which is interpreted in more detail in ESI Section 2.3. R_G is the radius of gyration of the nanoparticles and I_0 is the scattering intensity at 0 angle, both determined via the Guinier analysis of the SAXS data outlined in ESI Section 2.4. They were determined by extrapolation of the Guinier fit to $q = 0$. R_G is given as the average over the polymer concentrations of 0.5 – 2.5 mg mL^{-1}, while I_0 is given for c_{pol} = 2.5 mg mL^{-1}. N_{agg} is the weight-averaged number of polymer chains per particle calculated as described in ESI Section 1.2.5, given as average over the concentration range 0.5 – 2.5 mg mL^{-1}. The shape parameter α was a fixed input parameter for the form factor fits of p(J) and p(J-BD) equal to the slope in a log-log plot of the scattering intensity $I(q)$ against q between q = 0.8 – 1.2 nm^{-1} over the concentration range 0.5 – 2.5 mg mL^{-1}as outlined in ESI Section 2.4.

Table S1: Summary of the experimentally determined polymer properties using SEC, DLS and SAXS.

Polymer	$M_{n,th}$ (kg mol^{-1})	$Đ_{bb}$^{a} (-)	$Đ_{pol}$^{b} (-)	log(P) (-)	λ_{max} (nm)^c	R_H (nm)^c	R_G (nm)^d	I_0 (cm^{-1})^d	N_{agg} (-)	α (-)^d
p(J)	204	1.14	1.32	-2.42	652	11.8 ± 3.0	11.1 ± 0.8	0.21	1.1 ± 0.2	4.0 ± 0.2
p(J-BD)	177	1.14	1.27	-0.96	631	7.9 ± 0.5	9.3 ± 0.7	0.14	0.9 ± 0.2	3.2 ± 0.1
p(G-D)	25	1.14	-	-1.61	632	5.5 ± 2.4	3.8 ± 0.2	0.017	-	-
p(G-B)	27	1.14	-	-2.10	635	9.6 ± 3.7	8.5 ± 0.5	0.11	-	-

a) Determined by SEC in THF. b) Determined by SEC in DMF. c) Determined using DLS for p(J) and p(J-BD) in water and for p(G-D) and p(G-B) in PBS at pH = 7.4. d) Determined by SAXS in water.

2.2. Fluorescence Spectroscopy

The normalized Nile Red emission spectra for the different polymers either functionalized with Nile Red in the case of glucose-functionalized polymers, or physically encapsulating it in the case of Jeffamine-functionalized polymers, are shown in Figure S1. For p(J) and p(J-BD), the spectra are recorded in pure water at a polymer concentration of 1 mg mL^{-1} and a Nile Red concentration of 10 µM. For p(G-D) and p(G-B), the spectra are recorded in PBS at pH = 7.4 at a polymer concentration of 3 µM in PBS. The shape of the emission spectra is similar in all cases. Importantly, a clear difference is observed in the emission

maxima between polymers due to the solvatochromic nature of Nile Red. The emission maximum of λ = 652 nm observed for p(J) indicates that the Nile Red experiences a water-rich environment, confirming that p(J) does not form hydrophobic compartments. This is expected, as p(J) is completely functionalized with hydrophilic Jeffamine@M1000 grafts. The other 3 polymers show a significant blue-shift of their emission maxima compared to p(J), indicating that the Nile Red is experiences a significantly less hydrophilic environment. This indicates that these polymers are able to significantly shield Nile Red molecules from the bulk water, by providing hydrophobic compartments within their solution structures. This experiment therefore shows that the partial grafting of p(J-BD), p(G-D), and p(G-B) with hydrophobic grafts (dodecyl and BTA grafts) allows for the formation of hydrophobic compartments within the polymer particles in water. Moreover, the emission maxima of the Jeffamine-decorated p(J-BD) is similar to that of p(G-D), indicating that the shielding of Nile Red is not significantly influenced by the choice of hydrophilic graft between Jeffamine@M1000 and glucose. Interestingly, p(G-B) shows a slightly higher emission maximum at λ = 635 nm compared to λ = 632 nm for p(G-D), which indicates that the 5% BTA grafts of p(G-B) are less successful at shielding the Nile Red from the water-rich environment compared to the 15% dodecyl grafts of p(G-D).

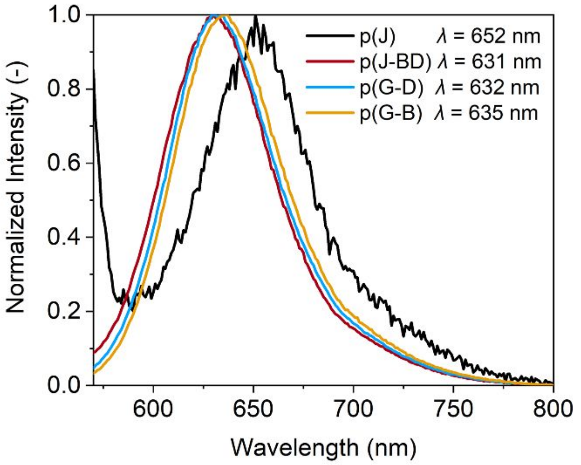

Figure S1: Normalized fluorescence emission spectra of Nile Red for the different polymers for the functionalized Nile Red (p(G-D) and p(G-B) in PBS or after physically encapsulating Nile Red (p(J) and p(J-BD)) in water.

2.3. Dynamic Light Scattering

The intensity and volume distributions of the hydrodynamic radius R_H as determined via DLS are shown in Figure S2A and S2B, respectively. DLS on p(J) and p(J-BD) were performed at a polymer concentration of 1 mg mL^{-1} and a temperature of 20.00 °C in pure water, whereas p(G-D) and p(G-B) were performed at a polymer concentration of 3 μM and a temperature of 25.00 °C in PBS. Importantly, DLS experiments on the different polymers in the aqueous media identify two size populations in the intensity distributions. Whereas the scattering contribution of the larger species is large in the intensity distribution, the corresponding volume distributions highlight that this larger species constitutes only a small fraction of the scattering species in solution. We therefore attribute the smaller majority species to our polymeric nanoparticles, and the larger minority species aggregates thereof in all cases. The hydrodynamic radii reported in Table S1 were derived from the intensity distribution. The hydrodynamic radii lie in the range of 5-12 nm. Without interpreting the exact numbers too much, it seems that p(J) forms larger particles than p(J-BD), which would be in good agreement with the formation of locally folded hydrophobic domains in p(J-BD) compared to a fully extended structure for p(J). Similarly, p(G-D) seems to form smaller particles compared to p(G-B). This is in line with the pronounced tendency of BTA to form aggregates which has also been observed in previous work.[21,22]

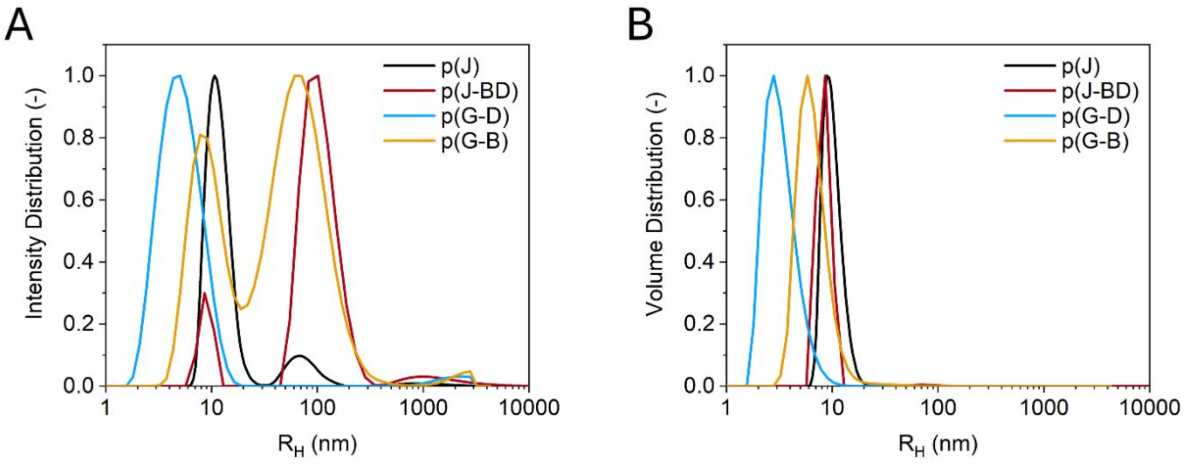

Figure S2: A) The intensity distribution and B) the corresponding volume distribution of the particle sizes present in aqueous solution of the different polymers as determined *via* DLS.

2.4. Small-angle X-ray Scattering

The scattering curves of the different polymers are shown in Figure S3. The curves of p(J) and p(J-BD) share similar features: An (approach to a horizontal) scattering plateau at low q (q < 0.1 nm^{-1}), a power law regime at intermediate q (0.1 < q < 0.6 nm^{-1}), and another power law regime at high q (0.6 < q < 1.5 nm^{-1}), until only incoherent background scattering is observed (q > 1.5 nm^{-1}). These scattering features are in good agreement with the theoretical scattering curves of semi-flexible polymer chains. Crucially, the presence of the intermediate and high q power law regime is consistent with semi-flexible polymers, and not with fully flexible polymers that can be described as generalized Gaussian coils (only a single power law scaling regime expected) or fully rigid polymers (expected power law scaling at intermediate q is q^{-1} instead). The horizontal plateau at low q corresponds to scattering originating from the entire particle. *Via* the Guinier analysis in this regime, shown in Figure S4, R_G and I_0 can be determined. The power law at intermediate q (0.1 < q < 0.6 nm^{-1}) is expected to be apparent in nature, as the chain dimensions of p(J) and p(J-BD) are too small for excluded volume effects to play a significant role in the folding behavior, as these effects should only become evident above L/l_k > 50 (see Table S2).[23] At high q (0.6 < q < 1.5 nm^{-1}), the observed power law changes once more as we probe even smaller distances. Here, the scattering originates from features smaller than the cross-sectional radius R_{cs} (particle surface), and the slope depends on the smoothness of the surface. A fractal dimension analysis in this regime yields the shape parameter α used in the form factor fitting of p(J) and p(J-BD). The curves of p(G-D) and p(G-B) share similar features to each other. In contrast to p(J) and p(J-BD), both show a scattering peak around q = 1 – 2 nm^{-1}. This feature can be indicative of core-shell formation, explored in the form factor fits shown in Figure S5. At low q, p(G-D) shows an approach to a horizontal scattering plateau (q < 0.1 nm^{-1}), in addition to an upturn indicative of scattering from aggregate structures. On the other hand, p(G-B) does not show a horizontal plateau, indicating that it forms larger structures in solution. Moreover, the scattering intensity at low q of p(G-B) is approximately 10 times higher than that of p(G-D), which is another indication that p(G-B) forms larger structures than p(G-D).

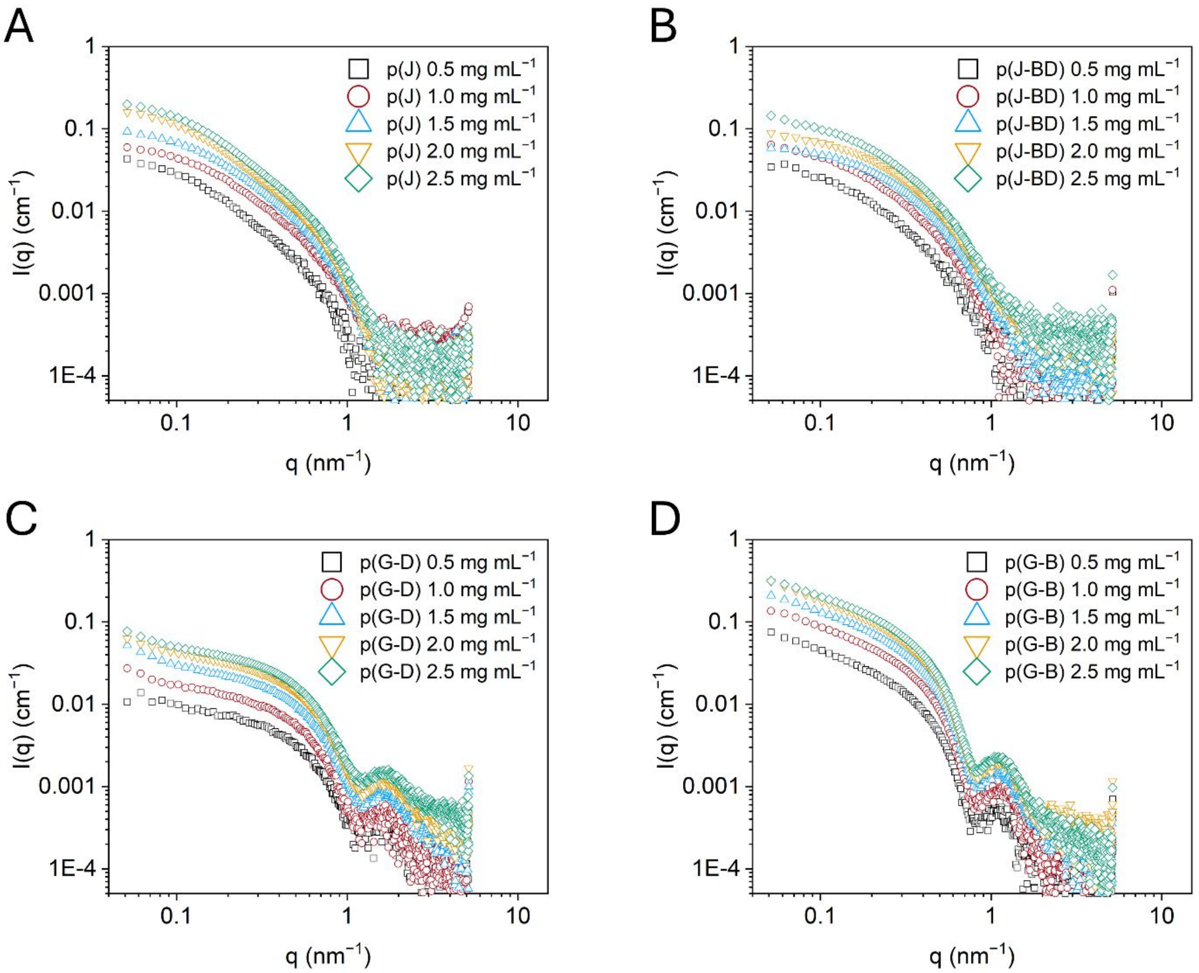

Figure S3: SAXS curves of the concentration series of A) p(J), B) p(J-BD), C) p(G-D), and D) p(G-B) in water measured at ESRF.[4]

The upturn at low q values is also visible in the Guinier plot (Figure S4A), comes from the presence of (a small amount of) larger aggregates, in agreement with the DLS data. R_G and I_0 are determined *via* the shape-independent Guinier analysis, summarized in Table S1. The aggregation number N_{agg} was determined from $I(0)_{exp}/I(0)_{th}$ for p(J) and p(J-BD) to assess whether the particles comprise a single polymer chain or more (Table S1). N_{agg} was found to be close to 1 for p(J) and p(J-BD), indicating that they are mostly present in solution as single-chain polymeric nanoparticles, with the number of larger aggregates being small in comparison. R_G is found to be 9 – 11 nm for p(J) and p(J-BD), again consistent with small nanoparticles in solution. There is a small decrease in R_G for p(J-BD) compared to p(J), consistent with a higher degree of chain compaction likely resulting from the formation of local hydrophobic domains in p(J-BD). Consistent with the larger hydrodynamic radius and the higher scattering intensity of p(G-B) compared to p(G-D), R_G = 8.5 nm for p(G-B) compared to 3.8 nm for p(G-D). A fractal dimension analysis to the power law regime observed at q = 0.8 – 1.2 nm^{-1} *via* a fractal dimension analysis yields the shape factor α as the observed power law, equal to 4.0 for p(J) and 3.2 for p(J-BD). This value is used as fixed parameter in the form factor fitting of the cross-section of these two polymers using a Boucher cylinder cross-section term (Figure S5).

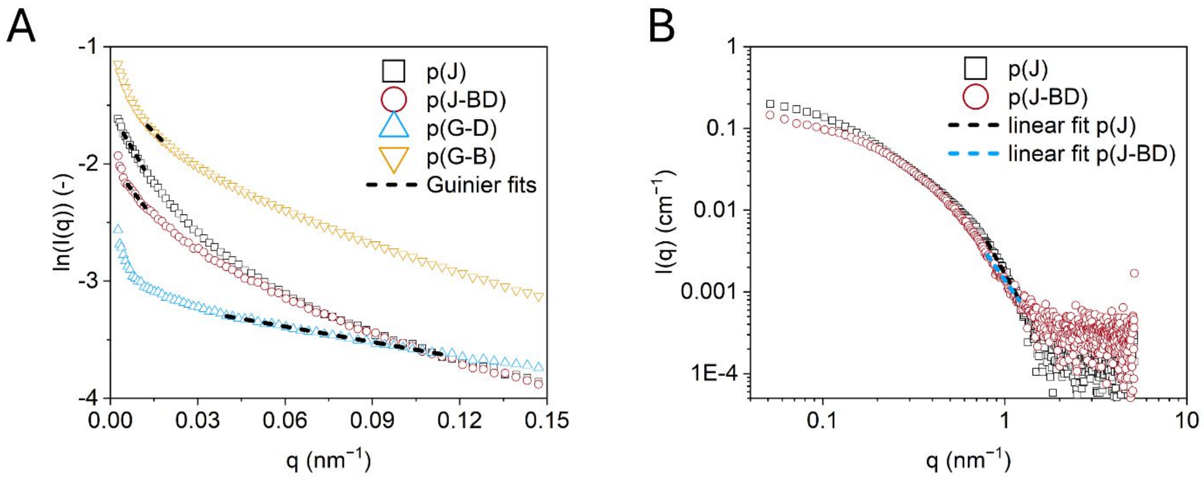

Figure S4: A) Guinier plots of the different polymers with linear fits used to determine R_G and I_0 *via* the shape-independent Guinier analysis. B) Fractal dimension analysis of p(J) and p(J-BD) performed at q = 0.8 – 1.2 nm^{-1}.

The form factor fits of the different polymers are shown in Figure S5. p(J) and p(J-BD) are fit as worm-like chains without excluded volume parameters, and p(G-D) and p(G-B) as core-shell ellipsoids with a power law to capture aggregate scattering, using a simultaneous fitting procedure over the concentration range (see ESI Section 1.2.5 for the full description of the model), yielding the global parameters summarized in Table S2. Different form factor models were explored. For the Jeffamine-based polymers, (core-shell) sphere, (core-shell) ellipsoid, and (core-shell) cylinder form factors could not properly fit the scattering curves. Additionally, a form factor model for generalized gaussian coils, which generally captures the scattering of fully flexible polymer chains well, did not agree with the experimental results. For the glucose-functionalized polymers, (core-shell) sphere, ellipsoids, and (core-shell) cylinder models did not work. See Tables S3 – S10 and Figure S7. In addition, both the generalized gaussian coil and worm-like chain models could not reproduce the scattering features properly. The current models shown here provided the best fit to the data. Due to the small dimensions of the polymer chains, the q-regime in which different parameters contribute to the scattering curve partially overlap. Therefore, multiple parameters show correlation. The current fits given in the text are stable, which was checked by changing one of the parameters to a fixed different value and optimizing the fit again. In all cases, the other parameters could not compensate for the change in the parameter value, resulting in fits with poorer goodness-of-fit compared to the fits given here. The form factors fits agree well with the experimental data. The form factor model of a worm-like chain used for the Jeffamine-based polymers agrees well with the global picture of the polymer chains obtained from the MD simulations, in which p(J) and p(J-BD) adopt globally stretched polymer conformations. The global parameters obtained for p(J) and p(J-BD) show similar dimensions, with one crucial difference: l_k is much smaller for p(J-BD) than for p(J), which is in good agreement with the extended nature of p(J) and the local formation of hydrophobic pockets for p(J-BD) that we obtained from the MD simulations. The form factor model used for the glucose-based polymers of a core-shell ellipsoid agrees well with the picture obtained from the MD simulations. The fit quality for p(G-D) is very good, with the power law reproducing the low q upturn, while the core-shell ellipsoid form factor reproduces the rest of the scattering features. The global parameters obtained from the simultaneous fitting procedure agrees well with the average of the individual fit ('-averaged'). The global parameters describe the p(G-D) nanoparticles as prolate core-shell spheroids, with the polar radius of the core 3 times larger than the equatorial radius, surrounded by a hydrophilic shell. The dimensions are all in the nm range, agreeing well with the formation of small core-shell nanoparticles comprising a single hydrophobic core. In good agreement with the other analyses, p(G-B) seems to form particles with larger dimensions (and/or different particle shapes including spherical and cylindrical objects may coexist), with a more pronounced prolate character owing to a larger R_{polar} compared to R_{eq}.

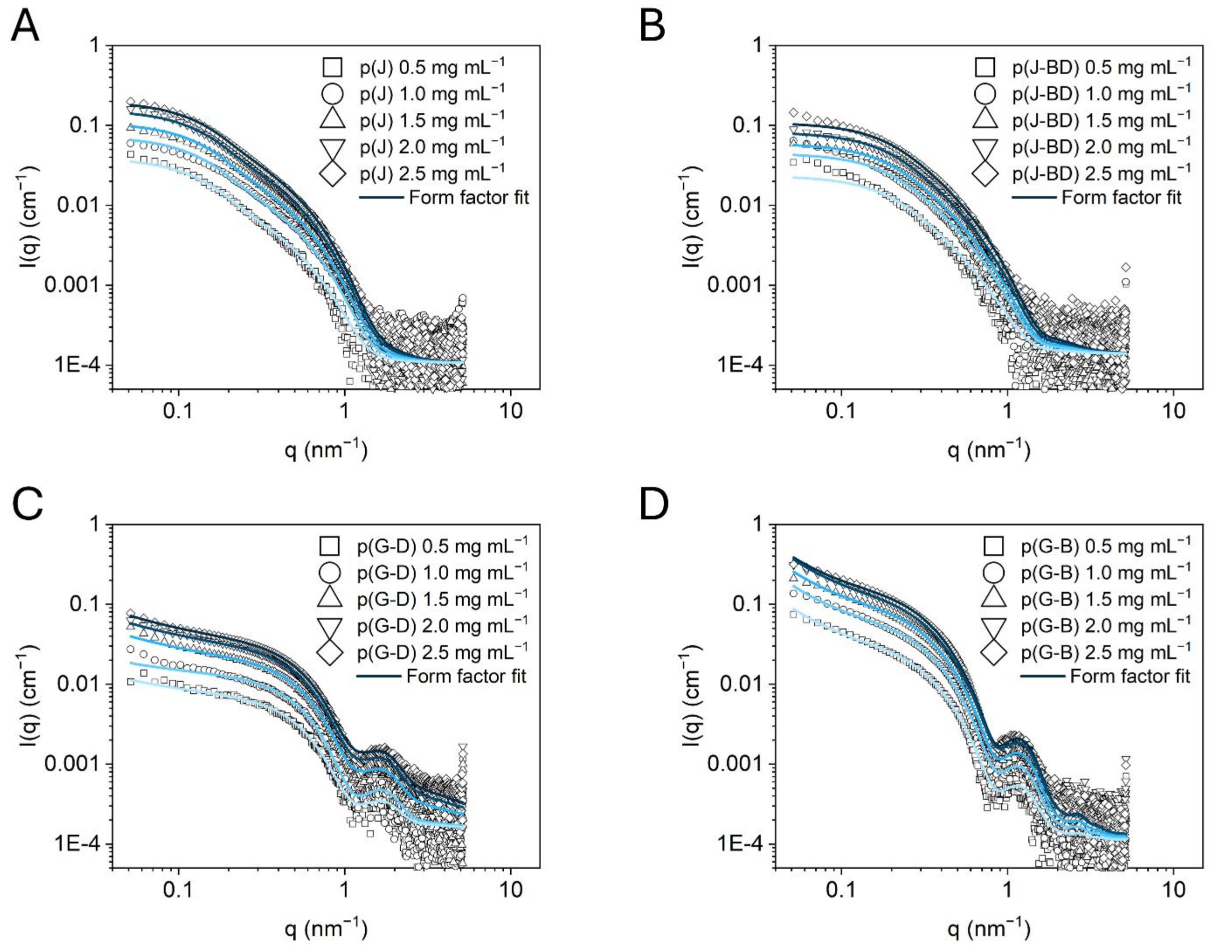

Figure S5: Simultaneous form factor fits of a worm-like chain without excluded volume interactions with global parameters for A) p(J) and B) p(J-BD). Simultaneous form factor fits of an additive core-shell ellipsoid and power law to fit aggregates with global parameters for C) p(G-D) and D) p(G-B). Fits were performed in SASfit,[5] see ESI Section 1.2.5. for a detailed explanation of the models.

Table S2: Overview of the global parameters resulting from the form factor fits using a worm-like chain without excluded volume interactions for P(J) and P(J-BD) and a core-shell model for p(G-D) and p(G-B) to the scattering curves of the different polymers.

Polymer	L (nm)	l_k (nm)	R_{cs} (nm)	σ_{cs}(-)	α (-)*	bg (cm^{-1})	χ^2 (-)
p(J)	55 ± 0	21 ± 0	2.1 ± 0.0	0.27 ± 0.00	4.0	0.0001 ± 0.000001	3.39
p(J-BD)	52 ± 1	7.0 ± 0.2	1.7 ± 0.1	0.24 ± 0.00	3.2	0.0001 ± 0.000001	3.94
Polymer	R_{polar} (nm)	R_{eq} (nm)	R_{shell} (nm)	R_G(nm)**	x^{-1} (-)	bg (cm^{-1})	χ^2 (-)
p(G-D)	3.35	1.00	2.00	250	0.86	0.0001	1.37
- averaged	3.31 ± 0.14	1.04 ± 0.02	1.90 ± 0.05	250	0.75 ± 0.04	0.0002 ± 0.0001	0.90 ± 0.13
p(G-B)	6.84	1.19	3.15	250	0.50	0.0001	5.74
- averaged	6.79 ± 0.16	1.19 ± 0.01	3.16 ± 0.04	250	0.49 ± 0.01	0.0001 ± 0.0001	5.2 ± 3.8

The following parameters obtained from the fitting procedure are listed: the length of the scattering objects L, the Kuhn length l_k, the cross-sectional radius R_{cs}, and the distribution width of the cross-sectional radius σ_{cs}, and the incoherent background level bg, in which chi-squared χ^2 is the error of the fit. The radius of gyration R_G, the polar radius of the ellipsoid R_{polar}, the equatorial radius of the ellipsoid R_{eq}, the thickness of the hydrophilic shell R_{shell} and the power law scaling x^{-1} (equal to the Flory exponent ν in the generalized Gaussian coil model). *α was determined in Figure S4B and fixed to the tabulated values during the fitting procedure. **R_G was fixed to the tabulated values during the fitting procedure to ensure the integrity of the power law fitting of the low-q upturn.

The Kratky plots of the polymers are shown in Figure S6. As expected for semi-flexible polymer chains with large grafts, the Jeffamine-based polymers show a peak in the Kratky plot, which indicates that their radii can be resolved beyond the background.[24,25] Importantly, the shape of the peak is different between the polymers. p(J) shows a broader peak than p(J-BD), indicating a more compact structure for the latter. Tailing is visible at higher q, indicating that the structures are not fully compacted, in good agreement with the previous analyses. Finally, around q = 0.25 nm^{-1}, p(J) shows an inflection point, which

is not discernable for p(J-BD). The inflection point corresponds to the transition from the Guinier regime to the observed power law regime at intermediate q, corresponding to a lower l_k for p(J-BD) compared to p(J). The Kratky plots for the glucose-based polymers are quite symmetric around their first peak, consistent with folded structures. The second peak is caused by the observed scattering peak in the log-log scattering curve that we assigned to the formation of a hydrophobic core.

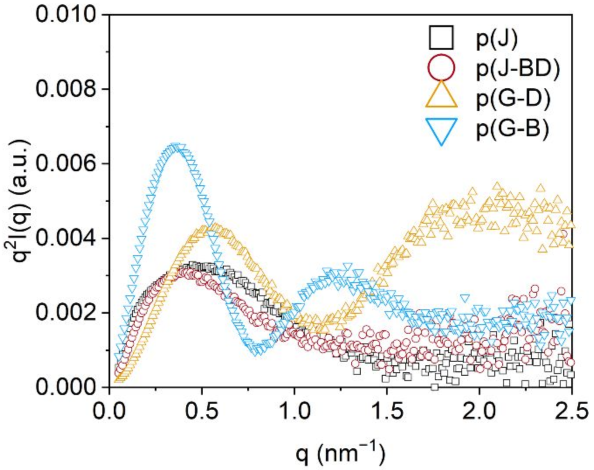

Figure S6: Kratky plots of the different polymers.

The fits corresponding to the alternative, unsuccessful models are given below (Figure S7). Fits were performed in SASfit,[5] see ESI Section 1.2.5. for a detailed explanation of the models. The following parameters obtained from the fitting procedure are listed: the sphere radius R, the length of the scattering objects L, the Kuhn length l_k, the cross-sectional radius R_{cs}, the radius of gyration R_G, the polar radius of the ellipsoid R_{polar}, the equatorial radius of the ellipsoid R_{eq}, the thickness of the hydrophilic shell R_{shell}, the Flory exponent ν , the incoherent background level bg, in which chi-squared χ^2 is the error of the fit. For the sphere model, see https://sasfit.org/#the-manual, page 57, section 3.1.1. For the core-shell sphere model, see https://sasfit.org/#the-manual, page59 , section 3.1.2. For the ellipsoid model, see https://sasfit.org/#the-manual, page 454, section 9.6. For the core-shell ellipsoid model, see https://sasfit.org/#the-manual, page 461, section 9.6.4. For the cylinder model, see see https://sasfit.org/#the-manual, page 475, section 9.7.6. For the core-shell cylinder model, see https://sasfit.org/#the-manual, page 476, section 9.7.7. For the generalized Gaussian chain model, see https://sasfit.org/#the-manual, page 416, section 9.4.4.1. For the worm-like chain model, see https://sasfit.org/#the-manual, page 365, section 9.2.4.5, for which the cross-section term $P_{cs}(q)$ was described as a Boucher cylinder cross-section see https://sasfit.org/#the-manual, page 343, section 9.2.2.5.

Table S3: Overview of the global parameters resulting from the form factor fits using a sphere model to the scattering curves of the different polymers.

Polymer	R (nm)	bg (cm^{-1})	χ^2 (-)
p(J)	4.0 ± 0.0	0.0003 ± 4e-6	1231
p(J-BD)	5.7 ± 0.0	0.0005 ± 76	61
p(G-D)	3.9 ± 0.0	0.0008 ± 3e-6	23
p(G-B)	6.1 ± 0.0	0.0005 ± 3e-6	254

Table S4: Overview of the global parameters resulting from the form factor fits using a core-shell sphere model to the scattering curves of the different polymers.

Polymer	R (nm)	R_{shell} (nm)	bg (cm^{-1})	χ^2 (-)
p(J)	5.9 ± 0.0	1.7 ± 0.0	0.0004 ± 4e-6	202
p(J-BD)	5.8 ± 0.0	0.1 ± 0.5	0.0005 ± 4e-6	61
p(G-D)	3.7 ± 0.0	1.3 ± 0.0	0.0007 ± 4e-6	22
p(G-B)	6.1 ± 0.0	0.0 ± 13	0.0005 ± 3e-6	255

Table S5: Overview of the global parameters resulting from the form factor fits using an ellipsoid model to the scattering curves of the different polymers.

Polymer	R_{polar} (nm)	R_{eq} (nm)	*bg* (cm^{-1})	χ^2 (-)
p(J)	45 ± 0	3.3 ± 0.0	0.0002 ± 4e-6	17
p(J-BD)	22 ± 0	3.5 ± 0.0	0.0003 ± 4e-6	3.4
p(G-D)	7.1 ± 0.0	3.0 ± 0.0	0.0007 ± 3e-6	12
p(G-B)	17 ± 0	4.3 ± 0.0	0.0004 ± 3e-6	56

Table S6: Overview of the global parameters resulting from the form factor fits using a core-shell ellipsoid model to the scattering curves of the different polymers.

Polymer	R_{polar} (nm)	R_{eq} (nm)	R_{shell} (nm)	*bg* (cm^{-1})	χ^2 (-)
p(J)	41 ± 0	0 ± 135	3.3 ± 11.9	0.0002 ± 4e-6	16
p(J-BD)	79 ± 1	3.2 ± 1.0	0 ± 1837	0.0003 ± 4e-6	9.5

Table S7: Overview of the global parameters resulting from the form factor fits using a cylinder model to the scattering curves of the different polymers.

Polymer	*L* (nm)	*R* (nm)	*bg* (cm^{-1})	χ^2 (-)
p(J)	1000*	3.0 ± 0.0	0.0002 ± 4e-6	19
p(J-BD)	34 ± 0	3.3 ± 0.0	0.0004 ± 4e-6	5.3
p(G-D)	10 ± 0	2.8 ± 0.0	0.0007 ± 3e-6	13
p(G-B)	26 ± 0	4.1 ± 0.0	0.0004 ± 3e-6	53

Table S8: Overview of the global parameters resulting from the form factor fits using a core-shell cylinder model to the scattering curves of the different polymers.

Polymer	*L* (nm)	*R* (nm)	R_{shell} (nm)	*bg* (cm^{-1})	χ^2 (-)
p(J)	1000*	3.0 ± 1.0	0 ± 65	0.0002 ± 5e-6	19
p(J-BD)	34 ± 0	3.3 ± 1.1	0 ± 72	0.0004 ± 6e-6	5.3
p(G-D)	14 ± 0	3.8 ± 0.0	0.7 ± 0.0	0.0006 ± 3e-6	9.4
p(G-B)	26 ± 0	0.9 ± 0.0	2.9 ± 0.0	0.0003 ± 3e-6	38

*L was fixed for the fit to converge.

Table S9: Overview of the global parameters resulting from the form factor fits using a generalized Gaussian coil model to the scattering curves of the different polymers.

Polymer	R_G(nm)	ν (-)	*bg* (cm^{-1})	χ^2 (-)
p(J)	9.5 ± 0.0	0.42 ± 9e-5	0.0002*	25
p(J-BD)	7.1 ± 0.0	0.36 ± 0.00	0.0001 ± 5e-6	5.7
p(G-D)	3.7 ± 0.0	0.22 ± 0.00	0.0006 ± 4e-6	12
p(G-B)	6.3 ± 0.0	0.25 ± 0.00	0.0003 ± 3e-6	99

**bg* was fixed to prevent a negative *bg*.

Table S10: Overview of the global parameters resulting from the form factor fits using a worm-like chain without excluded volume interactions model to the scattering curves of the different polymers.

Polymer	*L* (nm)	l_k (nm)	R_{cs} (nm)	α (-)*	*bg* (cm^{-1})	χ^2 (-)
p(G-D)	14 ± 0	6.5 ± 0.0	2.6 ± 0.0	4	0.0007 ± 3e-6	13
p(G-B)	17 ± 0	241 ± 1	4.0 ± 0.0	4	0.0004 ± 3e-6	43

*α was fixed to the value of 4, similar to p(J).

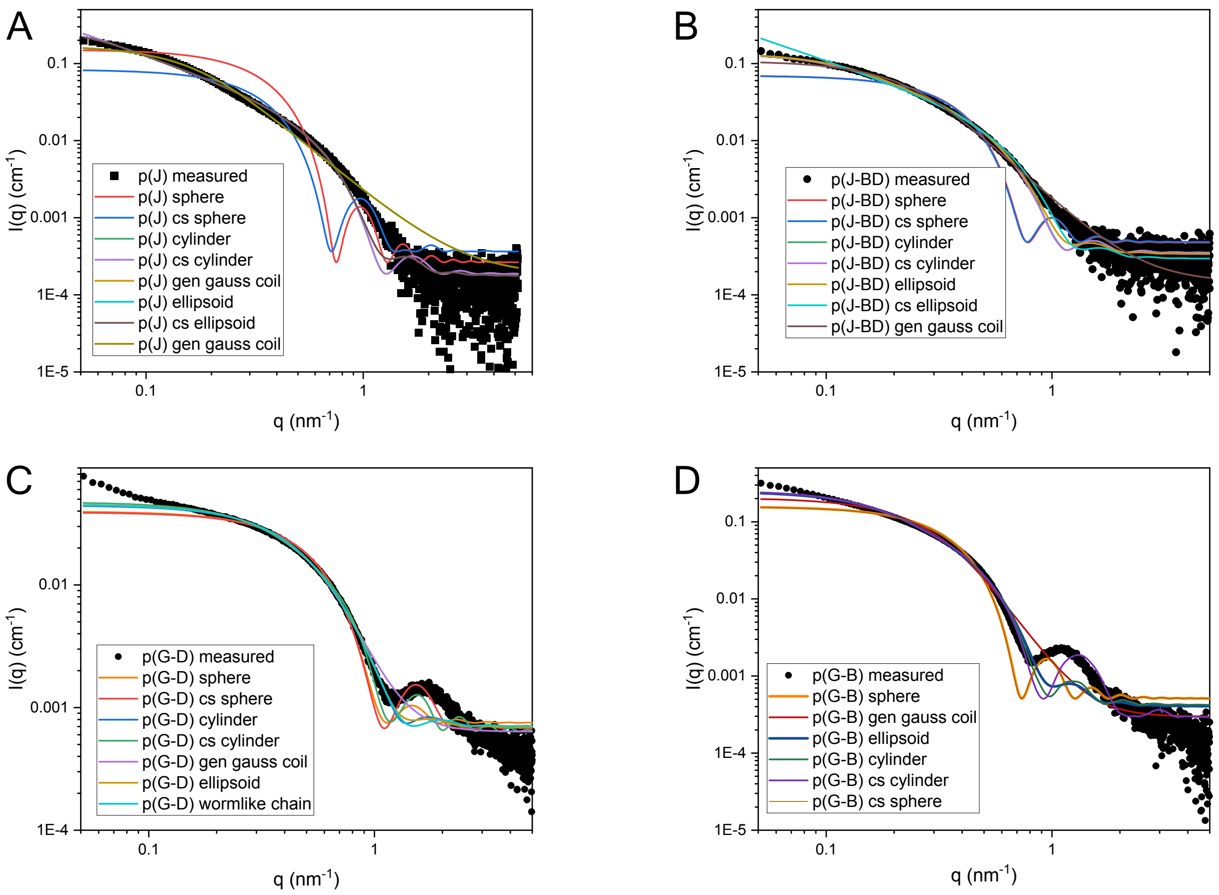

Figure S7: Different form factor fits to the experimental data of polymers (A) p(J), (B) p(J-BD), (C) p(G-D) and (D) p(G-B). None of the models used (sphere, cylinder, ellipsoid or general Gaussian coil), even not when using core shell (cs) models, fit well to the experimental data.

2.5. Results of MD simulations

A snapshot of p(J) I in its water box and with the 35,000 closest water molecules, and the final MD snapshots of all systems are shown in Figure S8. As mentioned in Section 1.2.6, three replicates were realized for each polymer and are distinguished by the Roman numerals I, II or III. The sequences are represented as color bars. The color legend is shown below the snapshots. First, a snapshot of p(J) I is shown inside its rectangular water box, all 797,257 water molecules being explicitly represented. The snapshot on its right shows p(J) I with its 35,000 closest water molecules, highlighting its important hydration. The hydrophilic p(J) systems remain extended, being more or less coiled. The p(J-*r*-BD) copolymers exhibit multiple hydrophobic clusters along the chain, leading to local folding and compaction but not to global collapse. All three replicates do not contain the same clusters, indicating that their formation depends on the “random” encounters between the hydrophobic groups during the simulations. These encounters are influenced by the primary structure, as, for example, the two dodecyl units isolated at the beginning of the sequence of monomers remain isolated in the 3D structures. The p(J-*b*-BD) copolymers all display an important coiling around the single hydrophobic core. The p(J-m*b*-BD) systems contain three distinct clusters, as pre-organized in their primary structure. The p(G) hydrophilic polymers exhibit a similar behavior as p(J), remaining extended coils. The glucose-based copolymers, however, are able to collapse into SCPNs. The p(G-D) and p(G-B) systems adopt similar conformations, core-shell structures, with the formation of one well-defined hydrophobic core surrounded by the glucose moieties. The formation of the core is not instantaneous, and the system may remain in partly-folded states for some time (see the final structure of p(G-D) I).

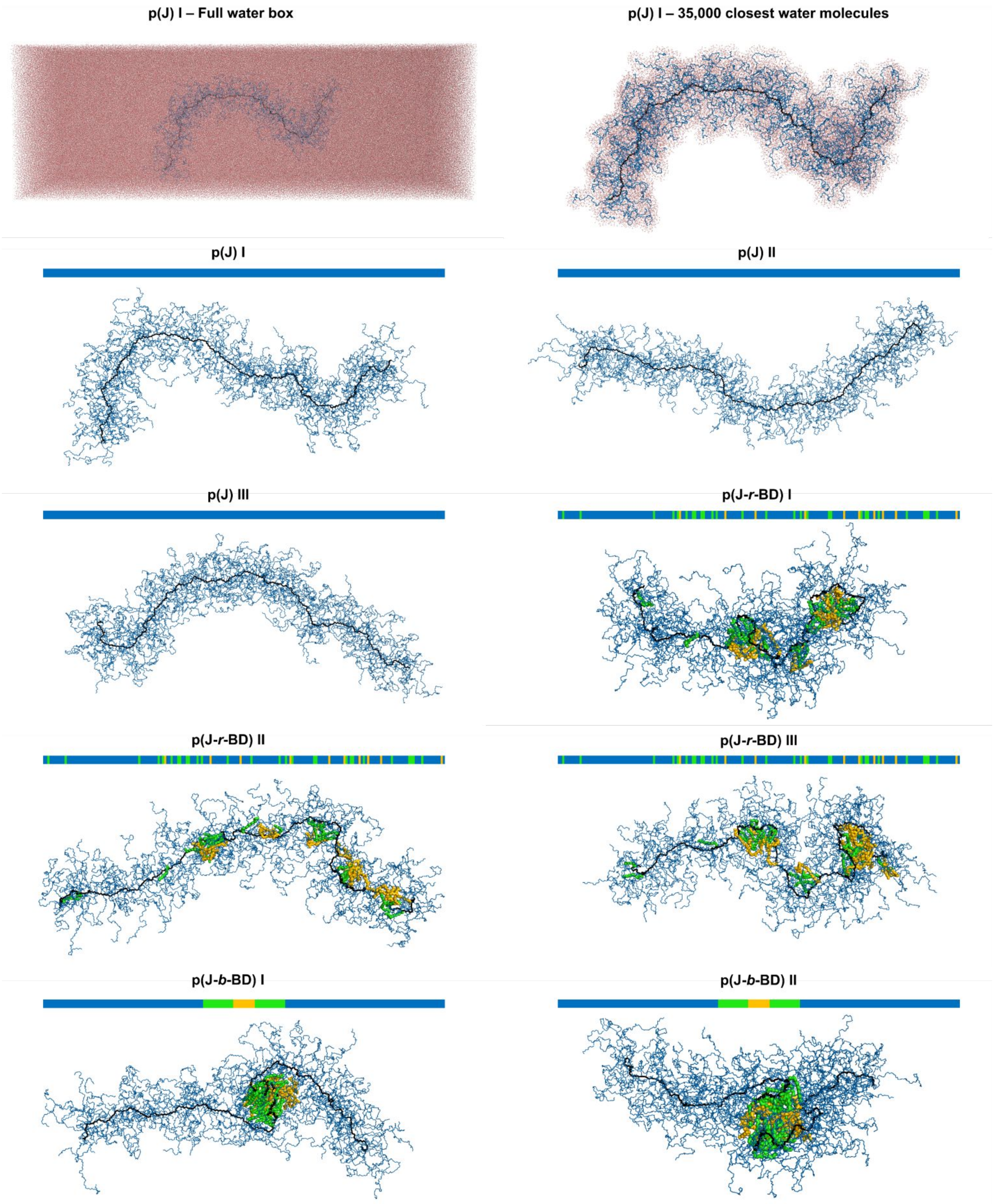
p(J) I – Full water box
p(J) I – 35,000 closest water molecules
p(J) I
p(J) II
p(J) III
p(J-r-BD) I
p(J-r-BD) II
p(J-r-BD) III
p(J-b-BD) I
p(J-b-BD) II

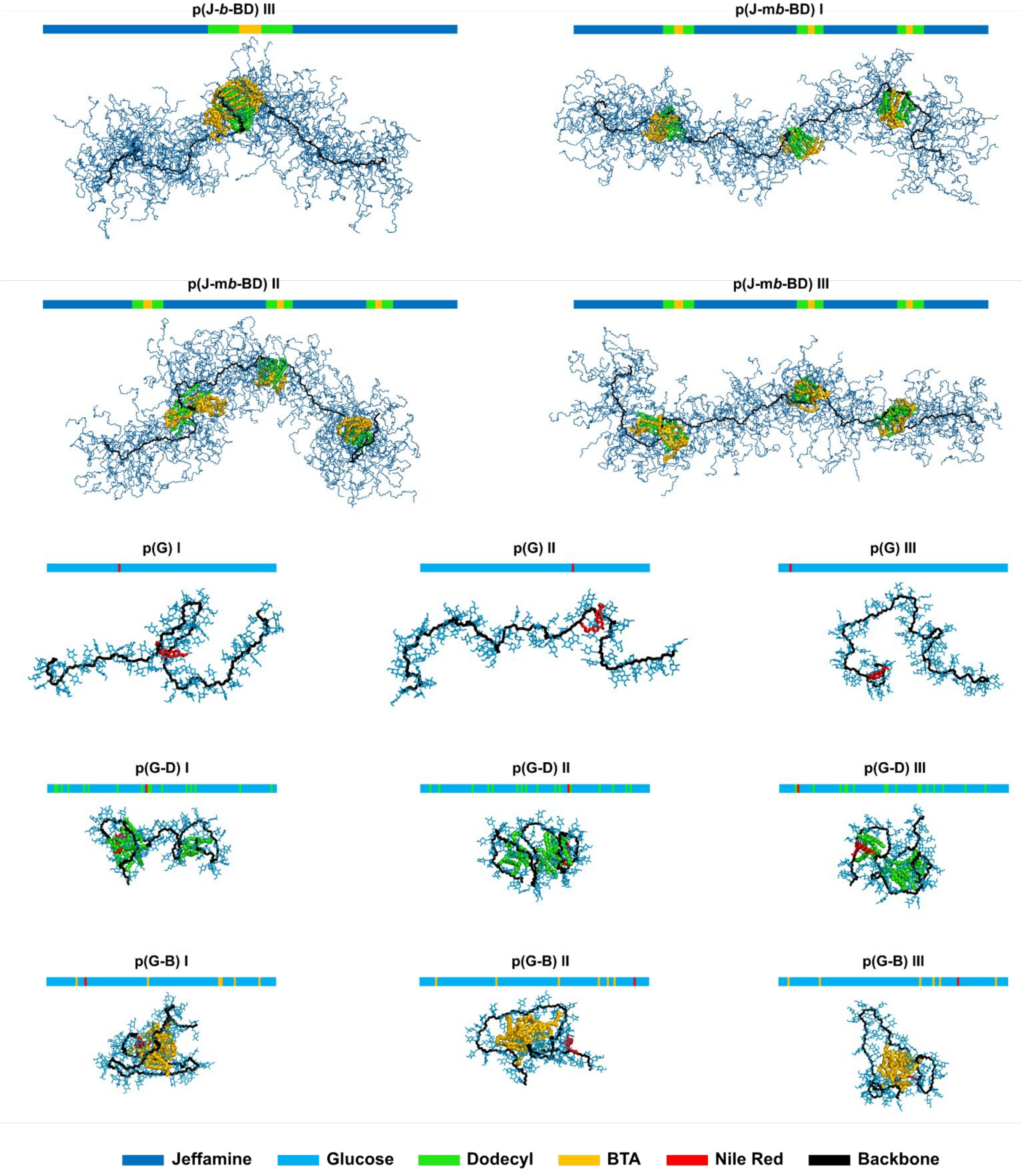

Figure S8: MD snapshots of the three replicates (I, II, III) for each polymer under study.

Additionally, the structures obtained by MD simulations were compared to those derived from SAXS experimental data. The χ^2 values, which assess the quality of the fit between the simulated and experimental curves, are shown for the different systems in the Table S11. The χ^2 values were also calculated excluding the first 50 points for the p(G-D) copolymers, because the upturn detected experimentally at the beginning of the spectrum (see Figure 2C in the main text) is caused by a population of aggregates. This upturn cannot be reproduced by our MD simulations, being performed on a small number of chains. For this reason, the χ^2 value displayed in the second column is more meaningful for p(G-D).

Table S11: Fit quality of the obtained SAXS curves from the MD simulations as compared to the experimental data.

Polymer	χ^2 (-)	χ^2 excluding first 50 data points (-)
p(J) – range 520 to 700 ns	2.7 ± 0.4	
p(J) – range 1820 to 2000 ns	2.4 ± 0.6	
p(J) – accelerated MD	3.2 ± 0.7	
p(J-BD) – range 520 to 700 ns	0.5 ± 0.2	
p(J-BD) – range 1820 to 2000 ns	0.8 ± 0.4	
p(J-BD) – accelerated MD	1.0 ± 0.3	
p(G-D) 1 chain	120 ± 7	53 ± 4
p(G-D) 2 chains	18 ± 3	3.7 ± 0.7
p(G-D) 3 chains	12 ± 1	3.1 ± 0.4

The evolution of the root-mean-square deviations (RMSD) values over time for all systems is shown in Figure S9. The RMSD values have converged at the end of the simulations, suggesting that the polymers have reached proper equilibration. Bigger fluctuations are observed for the p(G) systems, showing that these macromolecules remain flexible and do not stabilize into one specific conformation: they may coil and extend again over time. In some cases, the RMSD values converge to different values for different replicas of the same microstructure (see for example p(J-*r*-BD)). It reflects that, for a given microstructure, different conformations may be achieved. The data also show the evolution of RMSD values for the p(G-D) aggregates of two and three chains, which ran for 1920 and 1290 ns of aMD simulation, respectively (note that the accelerated protocol biases the sampling, meaning that these timescales cannot be considered as true or “physical” times).

Data on the RMSD for all systems

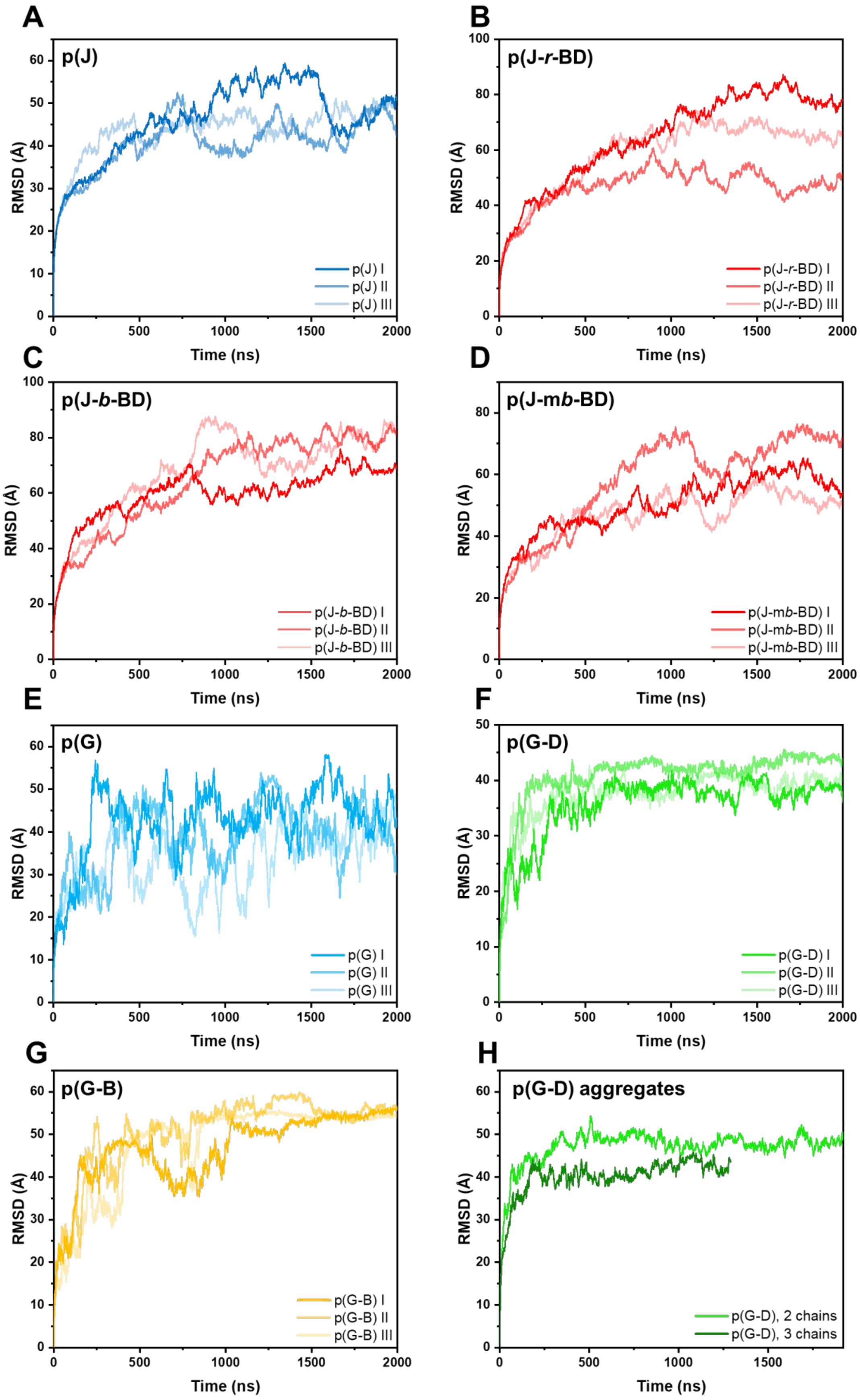

Figure S9: Evolution of the RMSD values with time for the (A) p(J), (B) p(J-*r*-BD), (C) p(J-*b*-BD), (D) p(J-m*b*-BD), (E) p(G), (F) p(G-D), (G) p(G-B) systems and (H) p(G-D) aggregates of two and three chains.

The data concerning the radii of gyration of the Jeffamine-based polymers are shown in Figure S10A-E. Figure S10A shows the evolution of R_G as a function of time for the three replicates of p(J) and p(J-*r*-BD). The R_G decreases in all cases, but it stabilizes at lower values for the copolymers. In some cases, all replicas of a given microstructure do not converge to the same value, indicating that the same microstructure can lead to different conformations, as mentioned above for the RMSD plots. The evolution of R_G as a function of time for the other microstructures of p(J-BD) is shown in Figure S8B-C. The box plot in Figure S8D shows the distribution of values measured over the last 400 ns of the simulation, showing clearly that all copolymers, no matter the sequence, adopt more compact structures than the hydrophilic p(J). The boxes are colored according to the system that they represent. The lines delimiting a box represent the first and third quartiles, whose values are annotated at the edges of the box. The line inside a box indicates the mean value. The error bar is given as mean ± 1.5 x standard deviation. Statistics are given in the table below, Figure S10E. As for all the other analyses, the statistics for a given system are computed on its three replicates.

The R_G values from the simulations of Jeffamine based systems are R_G = 9.5-10 nm for p(J) and $R_{G\,=}$ 6-8.5 nm for p(J-BD) when taking all different microstructures into account. Notably, the R_G values for the polymers with hydrophobic grafts are smaller than those of p(J) polymer. The observed trend is consistent with the experimentally derived R_G values from SAXS where we find higher values for p(J) compared to p(J-BD) (R_G = 11.1 nm for p(J) and R_G = 9.3 nm for p(J-BD)). Given the fact that experimental samples have a molar mass dispersity (both polymers) as well as a heterogeneity in microstructures (for p(J-BD)), the simulated R_G values are well in line with the experimentally derived ones.

Data on the radii of gyration for the Jeffamine-based systems

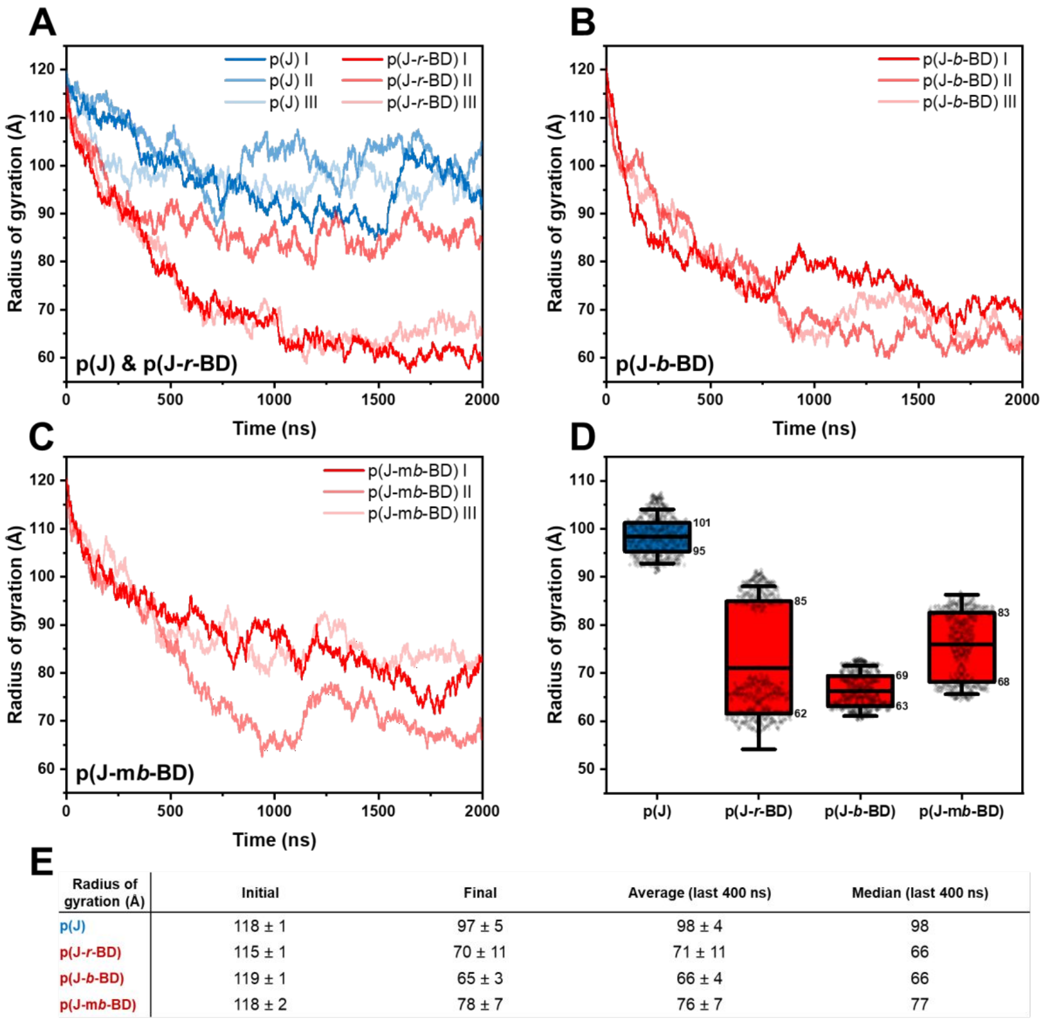

E

Radius of gyration (Å)	Initial	Final	Average (last 400 ns)	Median (last 400 ns)
p(J)	118 ± 1	97 ± 5	98 ± 4	98
p(J-*r*-BD)	115 ± 1	70 ± 11	71 ± 11	66
p(J-*b*-BD)	119 ± 1	65 ± 3	66 ± 4	66
p(J-m*b*-BD)	118 ± 2	78 ± 7	76 ± 7	77

Figure S10: Evolution of R_G for the (A) p(J) and p(J-*r*-BD), (B) p(J-*b*-BD) and (C) p(J-m*b*-BD) polymers during a 2000 ns simulation. (D) Overview of the R_G values obtained for the different polymers and microstructures averaged over the last 400 ns of the simulation. (E) Table summarizing the data.

The same data is provided for the glucose-based polymers in Figure S11A-E. The distributions of values for the p(G-D) and p(G-B) single-chain systems are narrower, as they form well-defined core-shell SCPNs. Data is also shown for p(G-D) aggregates of two or three chains, showing that they stabilize into slightly larger particles as the number of chains increases.

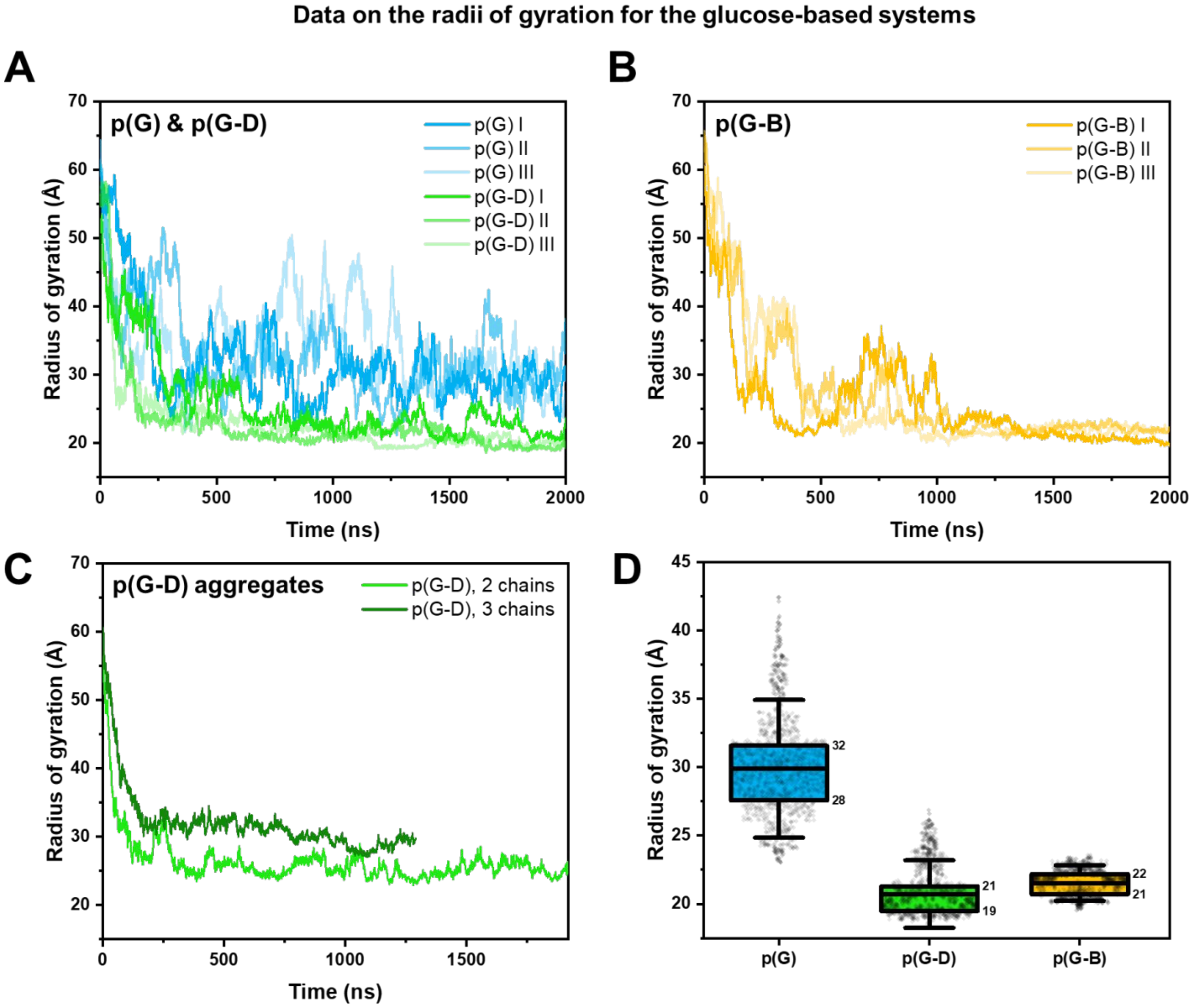

E

Radius of gyration (Å)	Initial	Final	Average (last 400 ns)	Median (last 400 ns)
p(G)	61 ± 3	32 ± 4	30 ± 3	30
p(G-D)	52 ± 2	21 ± 2	21 ± 2	20
p(G-B)	63 ± 2	21 ± 1	22 ± 1	22

Figure S11: Evolution of R_G for the (A) p(G) and p(G-D), (B) p(G-B) polymers during a 2000 ns simulation and (C) p(G-D) aggregates of two or three chains (1920 and 1290 ns of aMD simulation, respectively). (D) Overview of the R_G values obtained for the different single-chain systems, averaged over the last 400 ns of the simulation. (E) Table summarizing the data for the single-chain systems.

Figure S12A-B displays the data concerning the SASA of the Nile Red moiety, included in the glucose-based polymers. In Figure S12A, the boxes are colored according to the system that they represent (p(G) in blue, p(G-D) in green and p(G-B) in yellow). For each system, the box on the left represents the values measured over the first 400 ns and the box on the right, the values measured over the last 400 ns. In all cases, the SASA values are lower at the end of the simulations, indicating that Nile Red becomes less exposed to the solvent. The values are lower in the p(G-D) and p(G-B) systems, where the formation of core-shell SCPNs helps to bury the hydrophobic moieties. The lines delimiting a box represent the first and third quartiles, whose values are annotated at the edges of the box. The line inside a box indicates the mean value. The error bar is given as mean ± 1.5 x standard deviation. Statistics are given in the table below, Figure S12B.

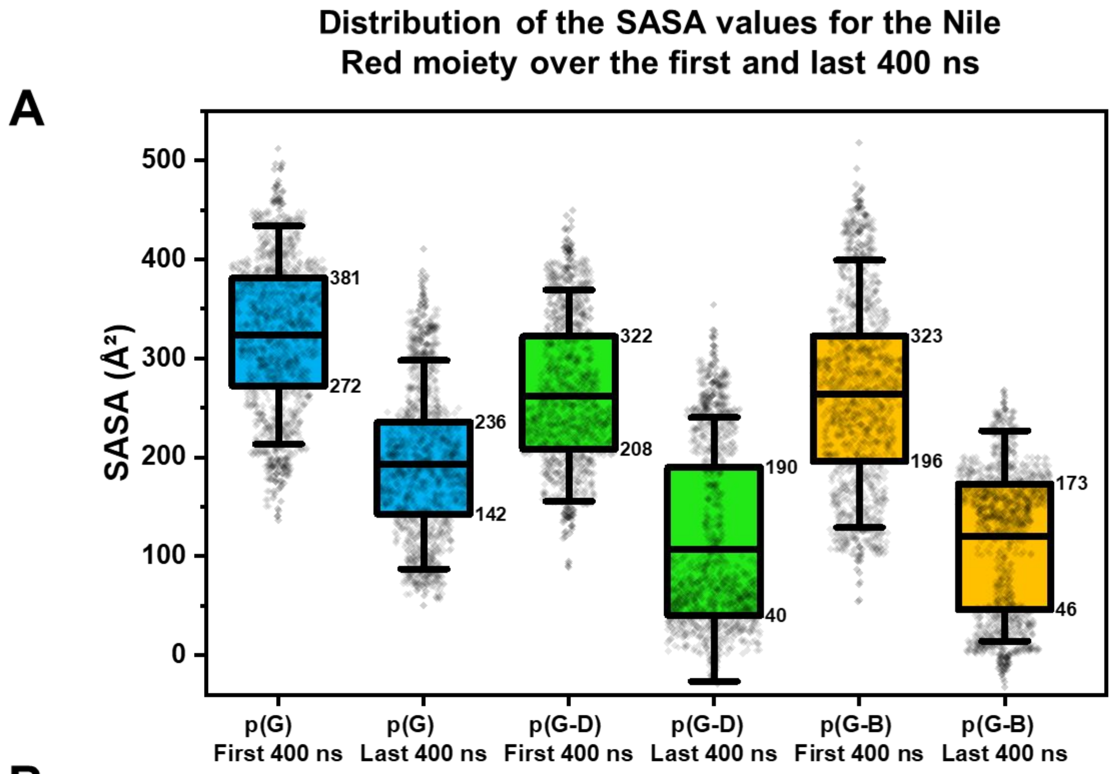

B

SASA Nile Red (Å²)	p(G) First 400 ns	p(G) Last 400 ns	p(G-D) First 400 ns	p(G-D) Last 400 ns	p(G-B) First 400 ns	p(G-B) Last 400 ns
**Average ± Std dev**	324 ± 73	193 ± 70	262 ± 71	107 ± 89	264 ± 90	121 ± 71
**Median**	331	188	249	69	266	144

Figure S12: (A) Distribution of the SASA values measured for the Nile Red moiety in the different polymers, averaged over the last 400 ns of the simulation. (B) Table summarizing the data.

Data on the backbone mobility is presented through root-mean-square fluctuations (RMSF) measurements in Figure S13A-C. The RMSF values measured over the first and last 400 ns for all systems are shown in Figure S13A and S13B, respectively. The lines delimiting a box represent the first and third quartiles. The line inside a box indicates the mean value. The error bar is given as mean ± 1.5 X standard deviation. The last line of the table displays the difference between the median value calculated over the last 400 ns and the one calculated over the first 400 ns. It shows a spectacular decrease for the p(G-D) and p(G-B) systems, meaning that their backbone became much less mobile when forming SCPNs. The lower decrease observed for the other systems can be attributed to their stabilization during the course of the simulation. This data is summarized in the table below, Figure S13C.

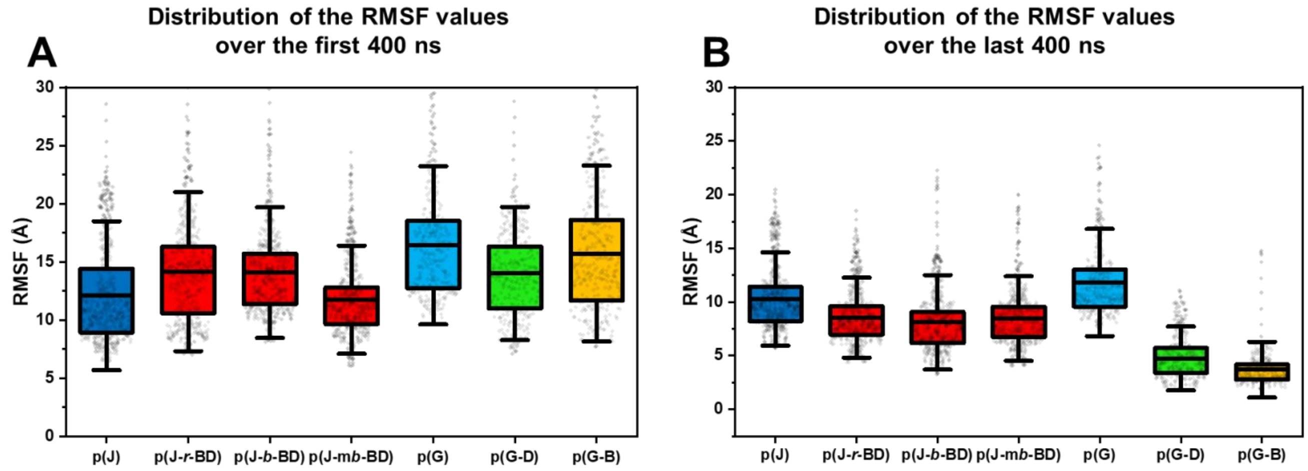

C

RMSF (Å)	p(J)	p(J-r-BD)	p(J-b-BD)	p(J-mb-BD)	p(G)	p(G-D)	p(G-B)
**First 400 ns**<br>**Average ± Std dev** *(median)*	12 ± 4 *(11)*	14 ± 5 *(14)*	14 ± 4 *(13)*	12 ± 3 *(11)*	16 ± 4 *(16)*	14 ± 4 *(14)*	16 ± 5 *(15)*
**Last 400 ns**<br>**Average ± Std dev** *(median)*	10 ± 3 *(10)*	9 ± 2 *(8)*	8 ± 3 *(8)*	8 ± 3 *(8)*	12 ± 3 *(11)*	5 ± 2 *(4)*	4 ± 2 *(3)*
**Delta median**	*- 1*	*- 6*	*- 5*	*- 3*	*- 5*	*- 10*	*- 12*

Figure S13: Distribution of the RMSF values over the (A) first and (B) last 400 ns of the simulations. (C) Table summarizing the data.

Data on the fluctuations of the backbone dihedral angles for the glucose-based systems, measured over the first and last 400 ns, is shown in Figure S14A-B. In Figure S14A, the three columns on the left indicate the percentage of the backbone dihedral angles which exhibited fluctuations superior than 20° around their mean value, for each system (p(G) in blue, p(G-D) in green and p(G-B) in yellow), over the first 400 ns. The columns on the right indicate this percentage for the last 400 ns. For the p(G-D) and p(G-B) copolymers, only around 10 % of the dihedral angles are still undergoing significant fluctuations during the last part of the simulation, showing that conformational sampling became limited as the SCPNs formed. This data is summarized in the table below, Figure S14B.

A

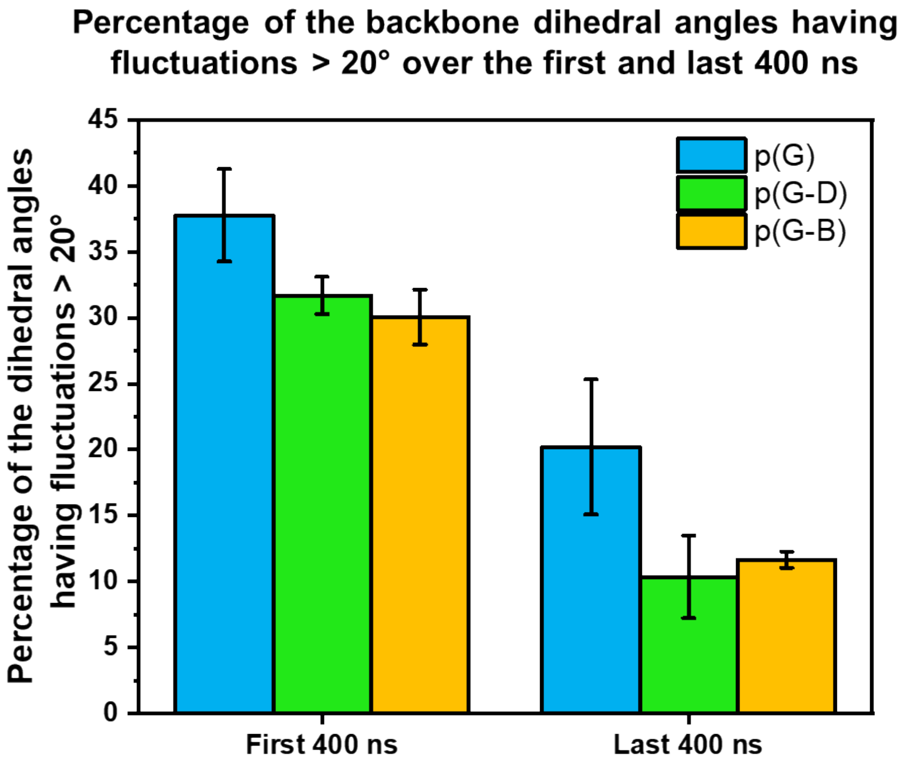

B

% of the dihedral angles with fluctuations > 20°	p(G)	p(G-D)	p(G-B)
First 400 ns - Average ± Std dev	37.8 ± 3.5	31.7 ± 1.4	30.0 ± 2.1
Last 400 ns - Average ± Std dev	20.2 ± 5.1	10.3 ± 3.1	11.7 ± 0.6

Figure S14: (A) Percentage of the backbone dihedral angles undergoing fluctuations superior than 20° in the glucose-based systems, over the first and last 400 ns. (B) Table summarizing the data.

Data on the intramolecular H-bonds in the glucose-based polymers is shown in Figure S15A-B. In Figure S15A, the boxes are colored according to the system that they represent (p(G) in blue, p(G-D) in green and p(G-B) in yellow). For each system, the box on the left *(right)* represents the values measured over the first *(last)* 400 ns. The lines delimiting a box represent the first and third quartiles, whose values are annotated at the edges of the box. The line inside a box indicates the mean value. The error bar is given as mean ± 1.5 X standard deviation. Data is summarized in the table below, in Figure S15B. The last line of the table displays the difference between the median value calculated over the last 400 ns and the one calculated over the first 400 ns. The number of intramolecular H-bonds increases significantly for the p(G-D) and p(G-B) copolymers, suggesting that these interactions contribute to stabilize the SCPNs.

A

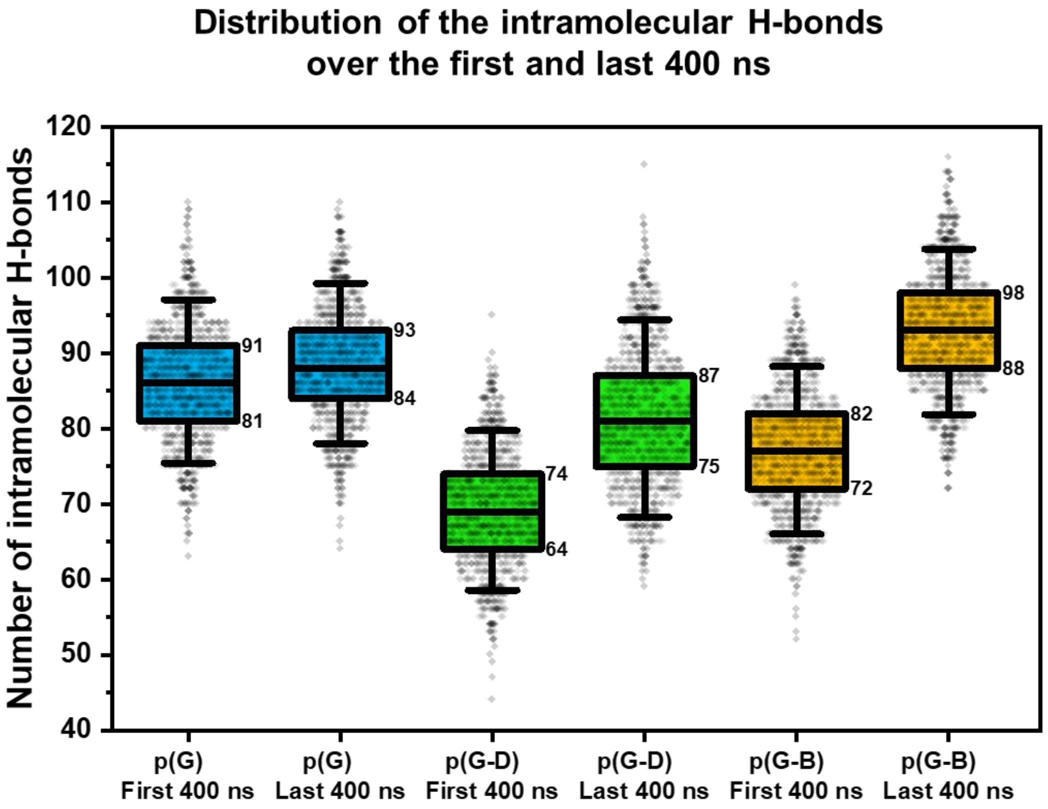

B

Number of intramolecular H-bonds	p(G) First 400 ns	p(G) Last 400 ns	p(G-D) First 400 ns	p(G-D) Last 400 ns	p(G-B) First 400 ns	p(G-B) Last 400 ns
Average ± Std dev	86 ± 7	89 ± 7	69 ± 7	81 ± 9	77 ± 7	93 ± 7
Median	86	88	69	81	77	93
Delta (median)	+ 2		+ 12		+ 16	

Figure S15: (A) Distribution of the number of intramolecular H-bonds in the different polymers, averaged over the first and last 400 ns of the simulation. (B) Table summarizing the data.

Figure S16A-B shows data on the hydrogen bonding interactions with the solvent. In Figure S16A, the graph displays the average number of H-bonds performed by each kind of side-chains with the solvent, per conformation, in all systems. The dodecyl chains are not represented, as they do not possess any H-bonds donor or acceptor. The Jeffamine grafts are, by far, the residues involved in the most frequent H-bonds with the solvent. The graph also suggests that the number of interactions with the solvent is not influenced by the system to which a graft belongs, i.e., a Jeffamine graft performs 18 H-bonds with the solvent, whether it is in the hydrophilic polymer p(J) or a copolymer p(J-BD). The data is summarized in the table below, Figure S16B.

A

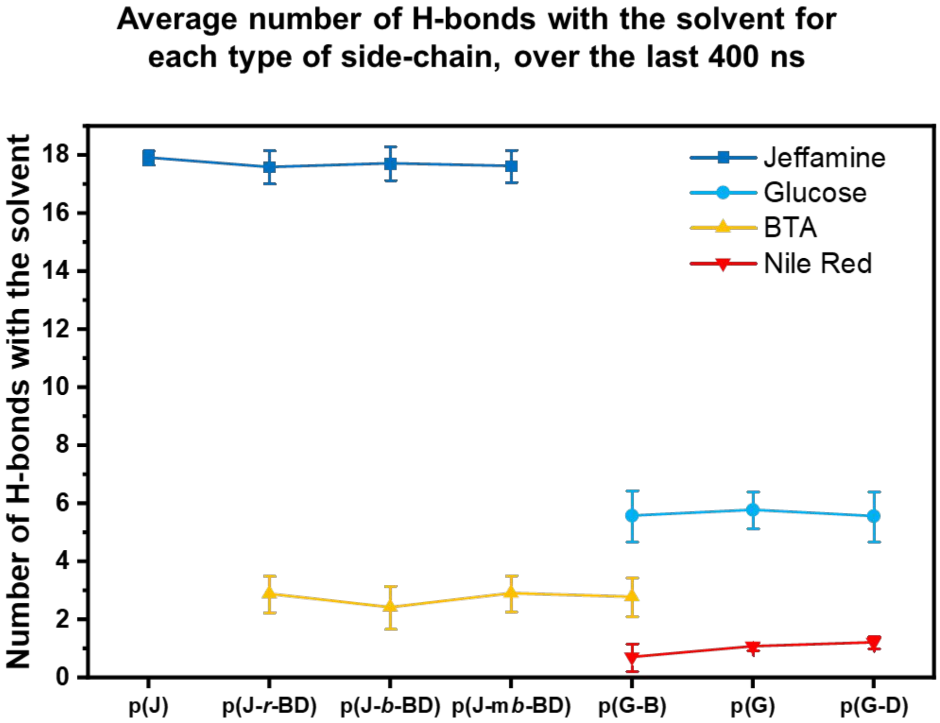

B

Number of H-bonds with the solvent per conformation	Jeffamine	Glucose	BTA	Nile Red
**p(J)**	18 ± 0	/	/	/
**p(J-*r*-BD)**	18 ± 1	/	3 ± 1	/
**p(J-*b*-BD)**	18 ± 1	/	2 ± 1	/
**p(J-m*b*-BD)**	18 ± 1	/	3 ± 1	/
**p(G-B)**	/	6 ± 1	3 ± 1	1 ± 0
**p(G)**	/	6 ± 1	/	1 ± 0
**p(G-D)**	/	6 ± 1	/	1 ± 0

Figure S16: (A) Number of hydrogen bonding interactions with the solvent per conformation, for all kinds of side-chains in each system, averaged over the last 400 ns. (B) Table summarizing the data.

Figure S17A displays the effect of the set of partial charges (AM1-BCC or RESP) on the conformations adopted by the p(J) polymers. The typical value of the charges computed on the atoms in the middle of a Jeffamine chain are represented in red and green for the oxygen and carbon atoms, respectively (not shown for hydrogen atoms). It seems that the charges of the heavy atoms computed with the AM1-BCC methodology are underestimated, the Jeffamine chains being weakly polar. It leads to the formation of compact, folded globules for these polymers, which is contradictory to experimental measurements. The RESP methodology leads to much better results, as the Jeffamine grafts remain hydrated and extended in the solvent, preventing compaction of the polymer. These results can be observed on Figure S17B for simple PEG chains, which where simulated during 200 ns in explicit water solvent. They tend to form compact structures when using AM1-BCC charges but remain extended when using RESP charges, as indicated by the evolution of the R_G over time. The last snapshot is shown for each set of partial charges (left: AM1-BCC, right: RESP). It shows that even commonly used parameters may be inaccurate, and may lead to very wrong predictions. Careful validation against experimental measurements is mandatory.

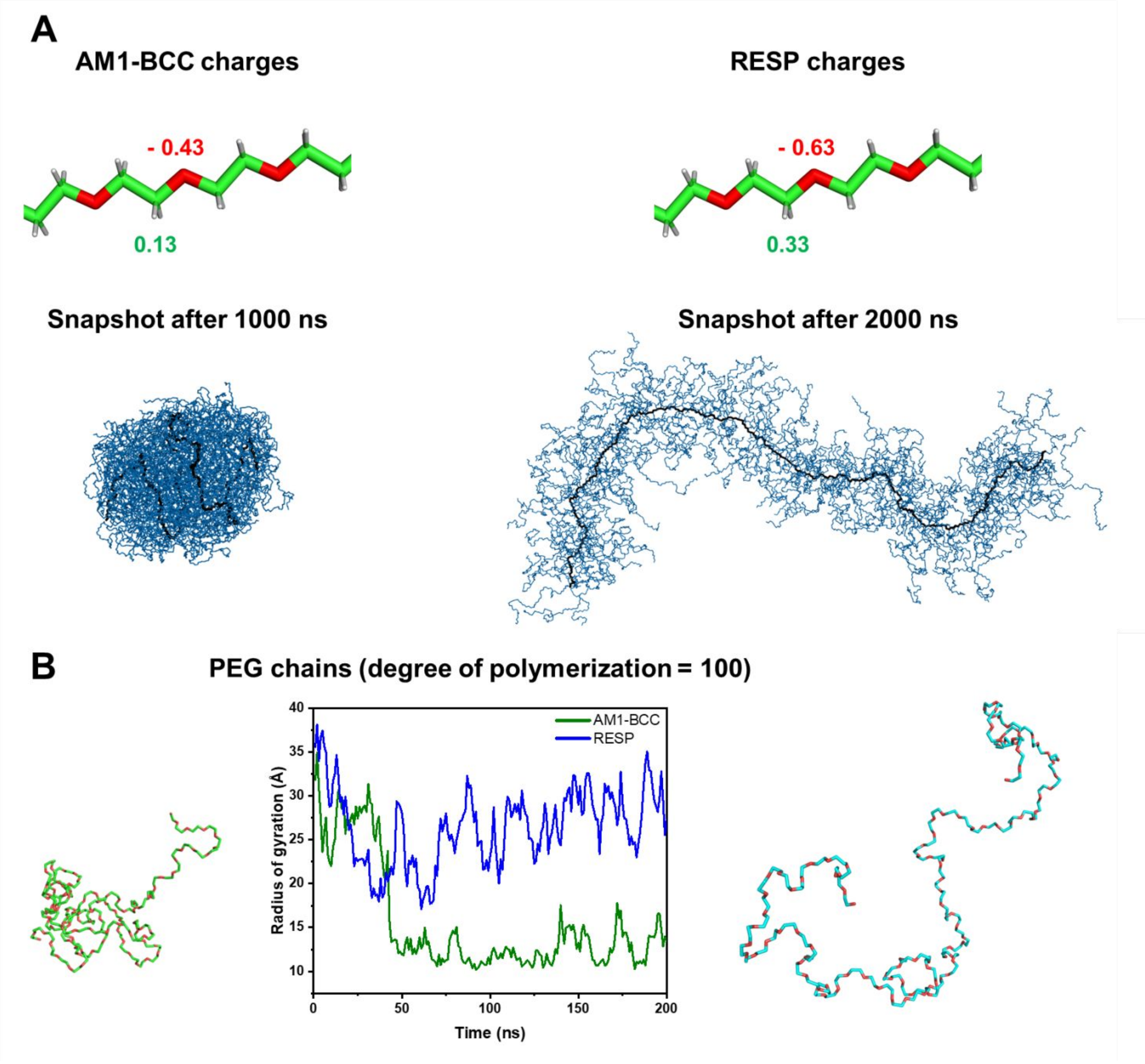

Figure S17: Investigation of the effect of the set of partial charges (AM1-BCC or RESP) on the conformations adopted by (A) the p(J) polymers and (B) simple PEG chains.

Data concerning the asphericity parameter, measured for all systems during the last 400 ns of the simulations, is shown in Figure S18A-B. In Figure S18A, the boxes are colored according to the system that they represent. The lines delimiting a box represent the first and third quartiles. The line inside a box indicates the mean value. The error bar is given as mean ± 1.5 X standard deviation. This data is summarized in the table, Figure S18B. The Jeffamine-based systems feature the higher values, as expected given their rod-like character. A small decrease can be observed for the p(J-BD) copolymers, which are slightly less extended due to the formation of the hydrophobic domains. The values measured for the p(G) polymers are more dispersed, highlighting their coiling and uncoiling over time. Finally, the p(G-D) and p(G-B) systems exhibit the values closest to zero, characterizing their folding into (non-perfectly) spherical SCPNs.

Data on the asphericity parameter

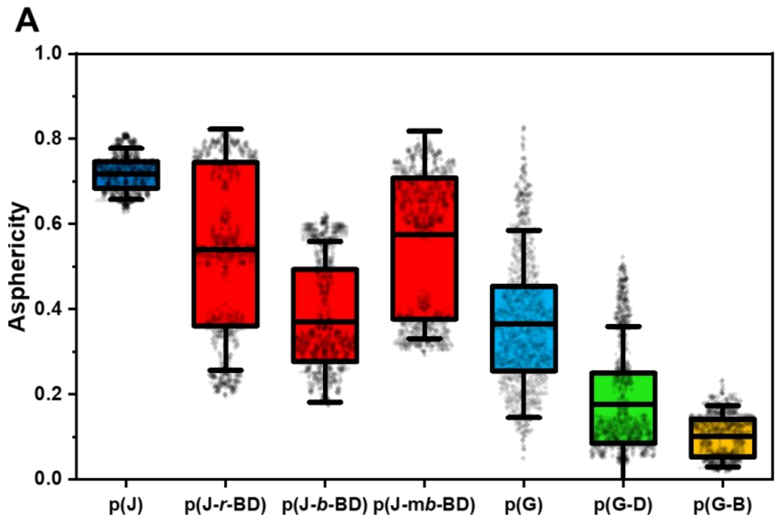

B

Asphericity	Mean ± Std dev
p(J)	0.72 ± 0.04
p(J-*r*-BD)	0.54 ± 0.19
p(J-*b*-BD)	0.37 ± 0.13
p(J-m*b*-BD)	0.57 ± 0.16
p(G)	0.37 ± 0.15
p(G-D)	0.18 ± 0.13
p(G-B)	0.10 ± 0.05

Figure S18: (A) Distribution of the asphericity parameter of each system, averaged over the last 400 ns. (B) Table summarizing the data.

The data concerning the SASA values of the hydrophobic grafts (dodecyl and BTA) over the last 400 ns are presented in Figure S19A-B. In Figure S19A, the graph shows that the SASA values do not change much depending on the system, indicating that the shielding of the hydrophobic moieties is similar in the extended p(J-BD) chains and in the p(G-D) and p(G-B) SCPNs. The values are even slightly lower for the Jeffamine-based polymers, whose extended conformations do not lead to exposure of the hydrophobic units to the solvent, thanks to the nature of the Jeffamine grafts. The data are summarized in the table below, Figure S19B.

A

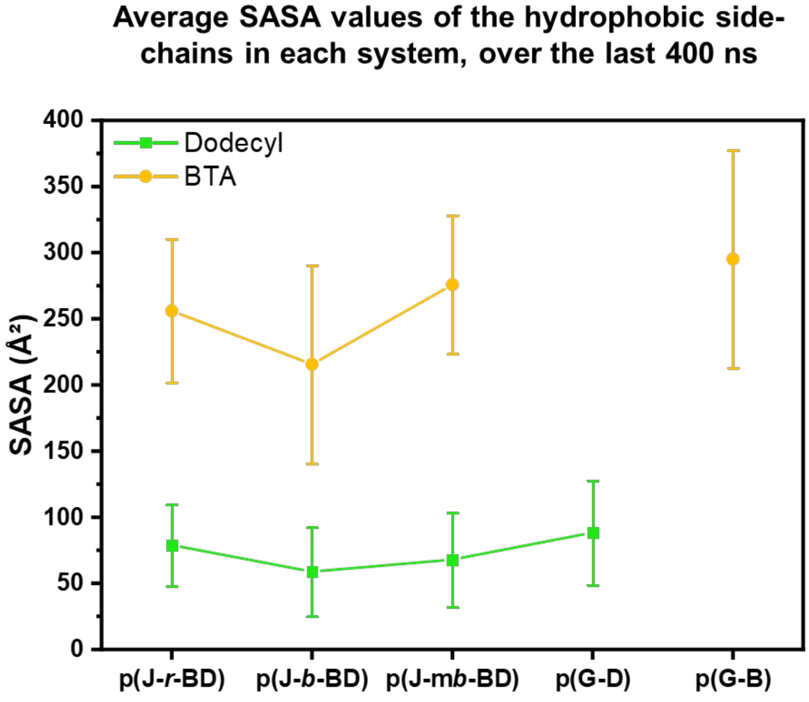

B

SASA (Å²)		p(J-*r*-BD)	p(J-*b*-BD)	p(J-m*b*-BD)	p(G-D)	p(G-B)
Dodecyl	Average ± Std dev	79 ± 31	59 ± 34	68 ± 36	88 ± 39	/
	Median	72	51	68	91	/
BTA	Average ± Std dev	256 ± 54	216 ± 75	276 ± 52	/	295 ± 82
	Median	253	204	262	/	303

Figure S19: (A) Average SASA value for each kind of hydrophobic moiety in all systems, measured over the last 400 ns. (B) Table summarizing the data.

Additional simulated SAXS curves are presented in Figure S20A-D. The curves were measured at the end of the simulation (range 1820-2000 ns) or generated from aMD simulations, for both p(J) and p(J-BD) systems. They show that the agreement with the experimental curves remains good over time, as confirmed by their χ^2 values, presented in Table S11.

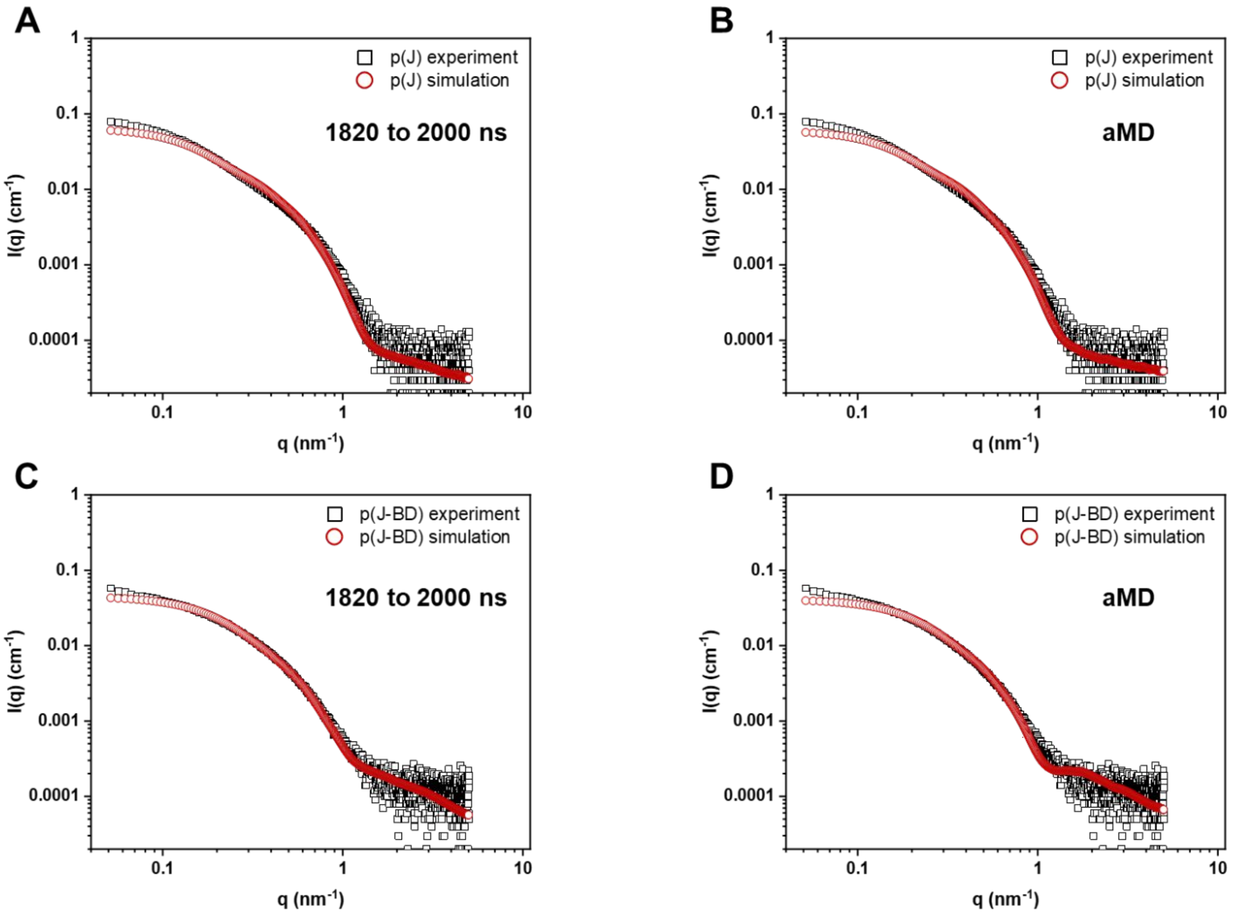

Figure S20: Experimental (black squares) and simulated (open circles) SAXS curves in water. (A) P(J) curve simulated in the range 1820 – 2000 ns. (B) P(J) curve generated from aMD simulations. (C) P(J-BD) curve simulated in the range 1820 – 2000 ns. (D) P(J-BD) curve generated from aMD simulations.

3. References

(1) Wijker, S.; Deng, L.; Eisenreich, F.; Voets, I. K.; Palmans, A. R. A. En Route to Stabilized Compact Conformations of Single-Chain Polymeric Nanoparticles in Complex Media. *Macromolecules* **2022**, *55* (14), 6220–6230. https://doi.org/10.1021/acs.macromol.2c00930.

(2) Deng, L.; Olea, A. R.; Ortiz-Perez, A.; Sun, B.; Wang, J.; Pujals, S.; Palmans, A. R. A.; Albertazzi, L. Imaging Diffusion and Stability of Single-Chain Polymeric Nanoparticles in a Multi-Gel Tumor-on-a-Chip Microfluidic Device. *Small Methods* **2024**, *2301072*, 1–12. https://doi.org/10.1002/smtd.202301072.

(3) J. R. Cooper, R. B. D. Release on the IAPWS Formulation for the Viscosity of Ordinary Water Substance. Berlin, Germany 2008.

(4) Sathyan, A.; Wijker, S. **2022**, DOI 10.15151/ESRF-ES-943025745.

(5) Kohlbrecher, J.; Breßler, I. Updates in SASfit for Fitting Analytical Expressions and Numerical Models to Small-Angle Scattering Patterns. *J. Appl. Crystallogr.* **2022**, *55* (6), 1677–1688. https://doi.org/10.1107/S1600576722009037.

(6) Jakalian, A.; Jack, D. B.; Bayly, C. I. Fast, Efficient Generation of High-quality Atomic Charges. AM1-BCC Model: II. Parameterization and Validation. *J. Comput. Chem.* **2002**, *23* (16), 1623–1641. https://doi.org/10.1002/jcc.10128.

(7) Hanwell, M. D.; Curtis, D. E.; Lonie, D. C.; Vandermeersch, T.; Zurek, E.; Hutchison, G. R. Avogadro: An Advanced Semantic Chemical Editor, Visualization, and Analysis Platform. *J. Cheminform.* **2012**, *4* (1), 17. https://doi.org/10.1186/1758-2946-4-17.

(8) Bayly, C. I.; Cieplak, P.; Cornell, W.; Kollman, P. A. A Well-Behaved Electrostatic Potential Based Method Using Charge Restraints for Deriving Atomic Charges: The RESP Model. *J. Phys. Chem.* **1993**, *97* (40), 10269–10280. https://doi.org/10.1021/j100142a004.

(9) Frisch, M. J.; Trucks, G. W.; Schlegel, H. B.; Scuseria, G. E.; Robb, M. A.; Cheeseman, J. R.; Scalmani, G.; Barone, V.; Petersson, G. A.; Nakatsuji, H.; Li, X.; Caricato, M.; Marenich, A. V.; Bloino, J.; Janesko, B. G.; Gomperts, R.; Mennucci, B.; Hratchian, H. P.; Ortiz, J. V.; Izmaylov, A. F.; Sonnenberg, J. L.; Williams-Young, D.; Ding, F.; Lipparini, F.; Egidi, F.; Goings, J.; Peng, B.; Petrone, A.; Henderson, T.; Ranasinghe, D.; Zakrzewski, V. G.; Gao, J.; Rega, N.; Zheng, G.; Liang, W.; Hada, M.; Ehara, M.; Toyota, K.; Fukuda, R.; Hasegawa, J.; Ishida, M.; Nakajima, T.; Honda, Y.; Kitao, O.; Nakai, H.; Vreven, T.; Throssell, K.; Montgomery, J. A. J.; Peralta, J. E.; Ogliaro, F.; Bearpark, M. J.; Heyd, J. J.; Brothers, E. N.; Kudin, K. N.; Staroverov, V. N.; Keith, T. A.; Kobayashi, R.; Normand, J.; Raghavachari, K.; Rendell, A. P.; Burant, J. C.; Iyengar, S. S.; Tomasi, J.; Cossi, M.; Millam, J. M.; Klene, M.; Adamo, C.; Cammi, R.; Ochterski, J. W.; Martin, R. L.; Morokuma, K.; Farkas, O.; Foresman, J. B.; Fox, D. J. *Gaussian 16, Revision A.03*; Gaussian Inc., Wallingford CT, 2016.

(10) Bertuzzi, D. L.; Becher, T. B.; Capreti, N. M. R.; Amorim, J.; Jurberg, I. D.; Megiatto, J. D.; Ornelas, C. General Protocol to Obtain D-Glucosamine from Biomass Residues: Shrimp Shells, Cicada Sloughs and Cockroaches. *Glob. Challenges* **2018**, *2* (11). https://doi.org/10.1002/gch2.201800046.

(11) Wang, J.; Wolf, R. M.; Caldwell, J. W.; Kollman, P. A.; Case, D. A. Development and Testing of a General Amber Force Field. *J. Comput. Chem.* **2004**, *25* (9), 1157–1174. https://doi.org/10.1002/jcc.20035.

(12) Schrödinger. *The PyMOL Molecular Graphics System, Version 2.5.4*; LLC, Ed.; 2022.

(13) Izadi, S.; Onufriev, A. V. Accuracy Limit of Rigid 3-Point Water Models. *J. Chem. Phys.* **2016**, *145* (7). https://doi.org/10.1063/1.4960175.

(14) Hopkins, C. W.; Le Grand, S.; Walker, R. C.; Roitberg, A. E. Long-Time-Step Molecular Dynamics through Hydrogen Mass Repartitioning. *J. Chem. Theory Comput.* **2015**, *11* (4), 1864–1874. https://doi.org/10.1021/ct5010406.

(15) Salomon-Ferrer, R.; Götz, A. W.; Poole, D.; Le Grand, S.; Walker, R. C. Routine Microsecond Molecular Dynamics Simulations with AMBER on GPUs. 2. Explicit Solvent Particle Mesh Ewald. *J. Chem. Theory Comput.* **2013**, *9* (9), 3878–3888. https://doi.org/10.1021/ct400314y.

(16) Hamelberg, D.; Mongan, J.; McCammon, J. A. Accelerated Molecular Dynamics: A Promising and Efficient Simulation Method for Biomolecules. *J. Chem. Phys.* **2004**, *120* (24), 11919–11929. https://doi.org/10.1063/1.1755656.

(17) Roe, D. R.; Cheatham, T. E. PTRAJ and CPPTRAJ: Software for Processing and Analysis of Molecular Dynamics Trajectory Data. *J. Chem. Theory Comput.* **2013**, *9* (7), 3084–3095. https://doi.org/10.1021/ct400341p.

(18) Bishop Christopher. *Pattern Recognition and Machine Learning*; 2006.

(19) Blavatska; von Ferber; Holovatch. Shapes of Macromolecules in Good Solvents: Field Theoretical Renormalization Group Approach. *Condens. Matter Phys.* **2011**, *14* (3), 33701. https://doi.org/10.5488/CMP.14.33701.

(20) Svergun, D.; Barberato, C.; Koch, M. H. J. CRYSOL – a Program to Evaluate X-Ray Solution Scattering of Biological Macromolecules from Atomic Coordinates. *J. Appl. Crystallogr.* **1995**, *28* (6), 768–773.

https://doi.org/10.1107/S0021889895007047.
(21) ter Huurne, G. M.; de Windt, L. N. J.; Liu, Y.; Meijer, E. W.; Voets, I. K.; Palmans, A. R. A. Improving the Folding of Supramolecular Copolymers by Controlling the Assembly Pathway Complexity. *Macromolecules* **2017**, *50* (21), 8562–8569. https://doi.org/10.1021/acs.macromol.7b01769.
(22) Abdouni, Y.; Ter Huurne, G. M.; Yilmaz, G.; Monaco, A.; Redondo-Gómez, C.; Meijer, E. W.; Palmans, A. R. A.; Becer, C. R. Self-Assembled Multi- And Single-Chain Glyconanoparticles and Their Lectin Recognition. *Biomacromolecules* **2021**, *22* (2), 661–670. https://doi.org/10.1021/acs.biomac.0c01486.
(23) Norisuye, T.; Fujita, H. Excluded-Volume Effects in Dilute Polymer Solutions. XIII. Effects of Chain Stiffness. *Polym. J.* **1982**, *14* (2), 143–147. https://doi.org/10.1295/polymj.14.143.
(24) Nakamura, Y.; Norisuye, T. Scattering Function for Wormlike Chains with Finite Thickness. *J. Polym. Sci. Part B Polym. Phys.* **2004**, *42* (8), 1398–1407. https://doi.org/10.1002/polb.20026.
(25) Nakamura, Y.; Norisuye, T. *Soft Matter Characterization*; Borsali, R., Pecora, R., Eds.; Springer Netherlands: Dordrect, The Netherlands, 2008.
